# An Engineered Nano‐Vesicle Adjuvant Platform (ENAP) for Cytokine Delivery Enables a Novel Antigen‐Coordinated Vaccine Against *Helicobacter pylori*

**DOI:** 10.1002/jev2.70274

**Published:** 2026-04-02

**Authors:** Yinpan Shang, Xiran Zhang, Linwei Li, Xiaomin Yu, Lingbing Zeng, Yanli Cao, Ziwei Tao, Lu Shen, Shuaishuai Zhang, Chuangye Yang, Huizhen Tian, Ying Liang, Hanchen Liao, Xiaotian Huang, Qiong Liu

**Affiliations:** ^1^ Department of Laboratory Medicine, The First Affiliated Hospital, School of Basic Medical Sciences, Jiangxi Medical College Nanchang University Nanchang China; ^2^ Medical Experimental teaching center, School of Basic Medical sciences Jiangxi Medical College Nanchang University Nanchang China; ^3^ Queen Mary School, Jiangxi Medical College Nanchang University Nanchang China; ^4^ HuanKui Academy Nanchang University Nanchang China; ^5^ Key Laboratory of Prevention and Treatment of Cardiovascular and Cerebrovascular Diseases, Ministry of Education, School of Basic Medicine Gannan Medical University Ganzhou Jiangxi China

**Keywords:** cytokine delivery, engineered nano‐vesicles, *H. pylori*, mucosal immunity, vaccine adjuvant

## Abstract

Despite the considerable potential of *Helicobacter pylori* (*H. pylori*) vaccines, their clinical efficacy has been hampered by inadequate mucosal immunity and suboptimal Th1/Th17 polarization. To address this, we engineered a novel nano‐adjuvant system using LPS‐modified recombinant outer membrane vesicles (rOMVs) derived from *H. pylori* to function as a programmable cytokine presentation platform. This engineered nano‐vesicle adjuvant platform (ENAP) confers unique synergistic advantages, including efficient delivery of key immunomodulatory cytokines such as IL‐17A and IFN‐γ, and potent activation of antigen‐specific T‐cell immunity. Following immunization, the platform significantly enhanced antigen‐specific mucosal IgA and systemic IgG2c/IgG1 antibody responses. It further induced a pronounced Th1/Th17‐skewed cellular immune response, resulting in a substantial reduction in bacterial colonization in a protective challenge model. Collectively, our study proposes a versatile and customizable nanotechnology strategy for reprogramming local and systemic immunity through targeted cytokine delivery, offering a promising avenue for the development of next‐generation mucosal vaccine adjuvants against *H. pylori* and other pathogens.

## 1 Introduction

Epidemiologic studies show that untreated *Helicobacter pylori* (*H. pylori*) infection can progress in a stepwise manner to atrophic gastritis and intestinal metaplasia, ultimately culminating in gastric cancer, a trajectory known as the Correa cascade (Liao et al. 2025). Amid rising antibiotic resistance, vaccination is widely regarded as the most promising strategy to control *H. pylori* infection (Hasanzadeh Haghighi et al. 2024). However, vaccine development targeting chronic mucosal pathogens such as *H. pylori* is impeded by several core challenges, including immune evasion mediated by host immune modulation, limited induction of mucosal immunity, rational antigen selection and formulation, and achieving a favourable safety profile complemented by durable, broadly protective efficacy (Friedrich and Gerhard 2023). Conventional live‐attenuated vaccines are constrained by reversion to virulence, limiting clinical translatability, whereas inactivated vaccines often exhibit insufficient potency owing to limited penetration of the gastric mucus layer. Although recombinant protein subunit vaccines offer a favourable safety profile, they are limited by fundamental shortcomings in currently available adjuvant systems: aluminium salts (alum) drive a Th2‐skewed response that fails to clear intracellular bacteria that reside within the gastric mucosa, whereas cholera toxin (CT) and heat‐labile toxin (LT) elicit strong immunity but pose toxicity concerns (Zhang et al. 2024, Patry et al. 2019). More advanced Toll‐like receptor (TLR) agonists—such as cytosine‐phosphate‐guanine oligodeoxynucleotides (CpG‐ODN) elicit robust systemic immunity yet often fail to achieve a balanced local cellular and humoral response in the stomach, leading to immune polarization: excessive Th2 responses drive tissue fibrosis, whereas insufficient Th1/Th17 activity permits persistent colonization (Bode et al. 2011). This lack of precise immune modulation remains a central bottleneck, hindering decisive breakthroughs in *H. pylori* vaccine development for decades.

Bacterial outer membrane vesicles (OMVs) constitute a promising vaccine delivery platform capable of overcoming key barriers in mucosal vaccination. *H. pylori* OMVs measure approximately 20–200 nm in diameter. Their lipid‐bilayer architecture endows them with distinct biological functions. First, their nanoscale dimensions and membrane composition that recapitulate the parent bacterium enable efficient endocytic uptake by antigen‐presenting cells (APCs), including dendritic cells and macrophages (Song et al. 2020, Tian et al. 2021). Second, abundant pathogen‐associated molecular patterns (PAMPs) engage multiple signalling pathways—notably TLRs and NOD‐like receptors (NLRs)‐thereby eliciting robust T‐cell responses (Toyofuku et al. 2023). Third, high surface levels of the adhesins BabA/BabB mediate specific recognition of the Lewis b antigen on gastric epithelial cells, precisely anchoring OMVs at the pathogen's colonization niche (Li et al. 2024). Finally, endogenous urease converts urea to ammonia, buffering local acidity and facilitating penetration of vesicles into the mucosal lamina propria (Kunkalienkar et al. 2025). Collectively, these features confer dual functionality—as both a delivery vehicle and an adjuvant. However, the intrinsic pro‐inflammatory activity of native OMVs, particularly the risk of lipopolysaccharide (LPS)‐mediated cytokine storms and their limited functional tunability, with stochastically packaged antigens that complicate standardization, severely limits their translational potential (Dowling et al. 2016).

To unlock the full potential of OMVs, recent studies have implemented multidimensional engineering strategies to reprogram OMV functions: Yue et al. engineered E. coli to express a ClyA–antigen–mFc fusion protein under an arabinose‐inducible promoter. Oral delivery of these bacteria together with arabinose led to in situ production of antigen‐displaying OMVs, which crossed the intestinal epithelium, were internalized by dendritic cells, and elicited potent antitumor immunity (Yue et al. 2022). Jingang Liu et al. developed IL‐10‐loaded extracellular vesicles (EVs) and subsequently modified the EVs with galactose for targeted therapy of inflammatory bowel disease (Liu et al. 2023). Similarly, Taotao Tang et al. leveraged macrophage‐derived EVs to deliver IL‐10 for targeted treatment of acute kidney injury (AKI) (Tang et al. 2020). Despite advances in loading cytokines into nano‐vesicles for molecular therapeutics, their potential as integral components of vaccine adjuvant systems has been largely overlooked. OMVs exert natural adjuvant effects as nano‐vesicles while also leveraging the immunostimulatory potential of cytokines. Our research group has previously constructed *H. pylori* OMVs capable of inducing immunostimulatory responses and demonstrated their efficacy as carriers for delivering major antigen proteins in vaccines, with promising results (Liu et al. 2025). This success prompts the question: Could these OMVs also be utilized for cytokine delivery?

Here, we describe an engineered nano‐vesicle adjuvant platform (ENAP) based on genetically engineered OMVs. We first engineered *H. pylori* to yield low‐toxicity strains with modified LPS to abrogate immune evasion, and efficiently encapsulated therapeutic plasmids within the vesicular lumen to create a ‘cytokine command depot’, thereby establishing a modular platform with broad applicability. Using on‐demand predictive analytics powered by artificial intelligence (AI), we generated two distinct cytokine formulations encoding interleukin‐17A (IL‐17A) or interferon‐γ (IFN‐γ), respectively. Compared with cytokine‐free recombinant OMVs (rOMVs) and CT, cytokine‐loaded ENAP—used as adjuvants for *H. pylori* vaccination in mice—elicited stronger and more durable humoral and mucosal immune responses across antigens. Importantly, relative to conventional adjuvant systems, ENAP constitutes a paradigm‐shifting advance. Its modular plasmid payloads enable on‐demand tailoring of Th1/Th17 responses, overcoming the Th2 bias of alum and the toxicity risks associated with CT‐type adjuvants. Collectively, we established a potent, modular ENAP that enables customizable cytokine delivery. Critically, ENAP recasts adjuvants from passive immunostimulants into a localized microenvironment‐reprogramming system, creating spatiotemporally confined cytokine niches that synchronously amplify mucosal and Th1/Th17 immunity, thereby providing a new toolkit for eradicating *H. pylori* and combating other mucosal pathogens.

## 2 Materials and Methods

### 2.1 Materials

OptiPrep density gradient medium and CT adjuvant were purchased from Southern Biotech (D1556 and C8082). Purified mouse immunoglobulin standards (IgG, IgG1, IgG2c or IgA) were purchased from BioLegend (400102, 401402, 400302 and 20102). Goat anti‐mouse IgG, IgG1, IgG2c and IgA antibodies were purchased from Southern Biotech (M6898, M8770, and A4789). Anti‐IL‐17, anti‐IFN‐γ, anti‐IL‐12 (P40), anti‐IL‐4 and anti‐IL‐6 antibodies were purchased from BD Biosciences (559501, 551216, 551219, 559062 and 554400). FITC anti‐mouse CD4 antibody, PE anti‐mouse CD154 antibody, APC anti‐mouse IFN‐γ antibody and Pacific Blue anti‐mouse IL‐17A antibody were purchased from BioLegend (116004, 106505, 505810 and 506918). YeaRred Nucleic Acid Gel Stain was purchased from Yeasen (10202ES76). DNA molecular weight markers were purchased from Genstar (M030). TRIzol and PrimeScript RT Master Mix were purchased from Takara (9109, RR036A).

### 2.2 Cytokine Screening and Evaluation

Cytokine selection was conducted using a multi‐step, AI‐powered screening pipeline. An initial candidate pool was compiled from public databases (e.g., ImmPort, UniProt) and literature mining. Each cytokine was represented as a feature vector incorporating immunological function annotations, network topology metrics, and mucosal expression profiles. A weighted scoring system was applied to prioritize candidates based on three criteria: immunological efficacy (weight: 0.5), mucosal safety (0.2), and synergistic potential (0.3). Machine learning models—including Extreme Gradient Boosting (XGBoost) for immunogenicity prediction, Support Vector Machine (SVM) for safety classification, and Multilayer Perceptron (MLP) for synergy forecasting—were employed to generate individual and combinatorial scores. The top‐ranked cytokine candidates and pairs (e.g., IL‐17A + IFN‐γ) were selected according to the integrated priority score (P‐score) for subsequent experimental validation.

### 2.3 Bacterial Strains and Cell Lines

*H. pylori* strains were cultured in brain heart infusion (BHI) broth supplemented with 10% exosome‐depleted fetal bovine serum (FBS, Every green) at 37°C under microaerobic conditions (5% O2, 10% CO2 and 85% N2). *H. pylori* strain 7.13, a gerbil‐adapted strain derived from the clinical isolate B128, was kindly provided by Professor Yong Xie (The First Affiliated Hospital of Nanchang University). Suspensions of *H. pylori* Sydney Strain 1 (SS1) were prepared from fresh exponential growth phase bacteria for the challenge experiment. The *H. pylori* mutant strain ∆*lpxE* ∆*lpxF* ∆*futB* was constructed using a suicide plasmid‐based method. In short, a pRE112 plasmid containing the upstream and downstream homologous arms of the target gene for deletion and antibiotic resistance cassette fragments was constructed. The plasmid was then transformed into *H. pylori* strain 7.13 through electroporation. Then, the gene knockout strain was obtained by screening with the corresponding resistance agar plate. Finally, the *H. pylori* mutant strain ∆*lpxE* ∆*lpxF* ∆*futB* was obtained through three sequential rounds of antibiotic selection.

In this study, specific *H. pylori* strains were selected for distinct experimental purposes based on their unique properties: the mouse‐adapted strain SS1 was used for in vivo challenge to ensure a reliable infection model (Sutton et al. 2000); strain 7.13, previously validated for its potent adjuvant activity, was chosen to produce OMVs for constructing the cytokine‐loaded adjuvant platform (Song et al. 2020); and the well‐annotated reference strain 26695 was employed to prepare OMPs as coating antigens for ELISA, ensuring standardized and reproducible immunological assessment (Baik et al. 2004).

The human embryonic kidney (HEK) 293T cells and the human gastric mucosal epithelial (GES‐1) cells were obtained from the National Collection of Authenticated Cell Cultures (Shanghai, China). The HEK 293T cells were cultured in Dulbecco's modified Eagle's medium (DMEM, Solarbio) supplemented with 10% FBS (Gibco). The GES‐1 cells were cultured in Roswell Park Memorial Institute (RPMI) 1640 (Solarbio) supplemented with 10% FBS (Gibco). All cell lines were maintained at 37°C in a humidified incubator with 5% CO2.

### 2.4 Purification and Characteristics of OMVs

As mentioned earlier (Li et al. 2022), OMVs were isolated from the culture supernatant of *H. pylori* and its mutants by ultracentrifugation. Briefly, 1 L of exponential‐phase bacterial culture supernatant was collected following low‐speed centrifugation (8000 rpm, 0.5 h and 4°C) and filtered through a 0.45 µm Steritop bottle‐top filter (Millipore). Then, the vesicles in the filtrate were precipitated by ultracentrifugation (100,000 × g, 2 h and 4°C) and resuspended in phosphate buffered saline (PBS). The initially isolated OMVs were further purified by ultracentrifugation (overnight, 200,000 × g and 4°C) on a discontinuous OptiPrep density gradient (Southern Biotech) prepared in PBS. The obtained vesicles were gently washed three times with PBS and finally dissolved in 1 mL of PBS. The total protein concentration was measured by bicinchoninic acid (BCA) assay (New Cell & Molecular Biotech) to determine the OMVs yield of different mutants under the same culture conditions. The morphology of OMVs was observed by transmission electron microscopy (TEM) at 120 kV. The characterization results were supported by Beijing Zhongkebaice Technology Service Co., Ltd. All OMVs were purified and quantified three times.

### 2.5 Construction of Plasmids and Electroporation

The recombinant plasmids were constructed by a seamless cloning method dependent on T5 exonuclease. Total RNA was extracted from mouse intestinal tissue and reverse‐transcribed into cDNA. The genes encoding IL‐17A and IFN‐γ were then amplified by PCR using this cDNA as a template. Finally, the PCR‐amplified IL‐17A and IFN‐γ fragments were inserted into the linearized pIRES plasmid using seamless cloning to generate the final expression plasmids. The recombinant plasmids were directly loaded into pre‐formed OMVs via electroporation. These engineered OMVs served as a recombinant adjuvant. The study incorporated four distinct OMV‐based adjuvant formulations: (i) 10 µg of OMVs loaded with the IFN‐γ plasmid; (ii) 10 µg of OMVs loaded with the IL‐17A plasmid; (iii) a 1:1 mixture of 5 µg of IFN‐γ plasmid‐loaded OMVs and 5 µg of IL‐17A plasmid‐loaded OMVs (10 µg total); and (iv) 10 µg of OMVs loaded with an empty plasmid (control).

### 2.6 Quantitative Polymerase Chain Reaction and Quantitative Reverse‐Transcription Polymerase Chain Reaction

The plasmid content within OMVs was quantified by quantitative PCR, as previously described (Ho et al. 2015). The expression levels of cytokines in HEK‐293T cells, GES‐1 cells, and mouse gastric mucosa were quantified by qRT‐PCR. Total RNA was extracted using TRIzol (Takara), and 500 ng RNA was converted into cDNA using PrimeScript RT Master Mix (Takara). qRT‐PCR was performed on three independent biological replicates, using 100 ng of cDNA per reaction. The primers of IL‐17A and IFN‐γ were as follows: IL‐17A (forward, 5′‐CTCCAGAAGGCCCTCAGACTAC‐3′; reverse, 5′‐AGCTTTCCCTCCGCATTGACAC‐3′), IFN‐γ (forward, 5′‐CAGCAACAGCAAGGCGAAA‐3′; reverse, 5′‐CTTTTCCGCTTCCTGAGGCT‐3′). Plasmid DNA was used as a positive control and the relative cytokine expression level was calculated with the 2‐ΔΔCt method.

### 2.7 Ethics Statement

All animal experiments conducted in this study met the requirements of the animal welfare guidelines of Nanchang University. Animal experiments were performed in accordance with the guidelines of the Laboratory Animal Ethics Committee of The First Affiliated Hospital of Nanchang University (Approval No. CDYFY‐IACUC‐202305QR024). Every effort was made to minimize animal suffering during experiments.

### 2.8 Immunization and Challenge Experiments

Six‐week‐old female C57BL/6 mice were purchased from the Laboratory Animal Science Centre of Nanchang University, and all mice were acclimatized for one week prior to being randomly divided into 18 groups (*n* = 9 per group).

UreB and WCV were used as antigens and combined with wild‐type and LPS‐modified *H. pylori* OMVs with cytokines, or CT as adjuvants, to construct a recombinant *H. pylori* vaccine. At the same time, a control group receiving 200 µL of PBS only was included. In this setup, UreB, OMVs, and CT were suspended in 200 µL of PBS buffer. The *H. pylori* WCV consisted of 10^9^ inactivated bacterial cells. The immunization and challenge schedules are illustrated in Figure 2a. Specifically, mice received two oral immunizations on day 0 and day 30. The immunogens and dosages for each group are detailed in Table S1. Blood samples were collected via orbital sinus puncture on the day prior to the first immunization and on days 14, 28, 42, 56, 70, and 84 post‐immunization. Vaginal washes were collected by flushing the vaginal tract five times with 100 µL of PBS per wash. Fecal samples were weighed and resuspended in PBS at a ratio of 0.5 mL per 100 mg of fecal material. Subsequently, the soluble fractions of serum, fecal supernatants, and vaginal washes were obtained by centrifugation. On day 56 after the initial immunization, half of the mice in each group were euthanized to collect gastric mucosa, mesenteric lymph nodes (MLN), and splenic lymphocytes for cytokine analysis. On day 96 post‐immunization, all remaining mice were orally challenged with 10^9^ colony‐forming units (CFU) of *H. pylori* SS1 suspended in PBS containing 0.01% gelatin and were monitored until day 110. All mice were then euthanized, and gastric tissues were harvested for bacterial load and urease activity assessments. Additionally, spleen lymphocytes were isolated for flow cytometry analysis.

Similarly, we conducted similar experiments on gene knockout mice. The immunization and challenge schedules are shown in Figure 6b. Mice received oral immunizations on day 0 and day 30. Blood and vaginal wash samples were collected pre‐immunization and on day 56. Half of the mice were euthanized on day 56 for splenic lymphocyte analysis. The remaining mice were challenged with 10^9^ CFU of *H. pylori* SS1 on day 60 and monitored until day 90, after which gastric tissues were collected for bacterial load and urease activity assessment.

### 2.9 Determination of Immunoglobulin Content by ELISA

The levels of immunoglobulin in the serum, vaginal washes and fecal samples of mice after immunization were determined by ELISA to evaluate the immune response. The assay was performed as previously described (Liu et al. 2024). Briefly, 96‐well plates were coated overnight at 4°C with 1 µg of purified recombinant UreB or OMPs from *H. pylori* strain 26695 in sodium bicarbonate buffer (pH 9.6). In addition, purified mouse immunoglobulin (Ig) standards (IgG, IgG1, IgG2c or IgA; BioLegend) were used for multiple dilutions to construct a standard curve for quantifying the homotypes of each antibody. Finally, quantitative ELISA was conducted using biotinylated goat anti‐mouse IgG, IgG1, IgG2c and IgA (Southern Biotech) as secondary antibodies. The concentration of each antibody isotype was calculated from the corresponding standard curve. All ELISA experiments were repeated three times.

### 2.10 Cytokine Assay

To evaluate T‐cell immune responses, the secretion profiles of key cytokines (IFN‐γ, IL‐12p40, IL‐4, IL‐17, IL‐6) were analysed. The specific experimental protocol is as follows: 26 days after the booster immunization, the spleen lymphocytes and MLN cells of mice in each group were aseptically isolated. After adherence, cells were stimulated with *H. pylori* 26695 OMPs (6 µg/mL) for 24 h. Supernatants were then collected for analysis. The secretion levels of IL‐4 (Th2 characteristic factor), IL‐6 (inflammatory marker), IFN‐γ and IL‐12p40 (Th1 polarization key factor), IL‐17 (Th17 characteristic factor) were quantitatively detected strictly in accordance with the instructions using high‐sensitivity ELISA. This experimental design can comprehensively assess the differentiation characteristics of T‐cell subsets induced by vaccines and potential inflammatory risks by comparing the cytokine response patterns of different cell sources (systemic immunity and mucosal immunity).

### 2.11 Opsonization Assay

An opsonophagocytosis assay was conducted following an established protocol with modifications for *H. pylori* (Naess et al. 1999). In brief, peritoneal macrophages were harvested from C57BL/6 mice via peritoneal lavage using ice‑cold PBS. Cells were pelleted by centrifugation and resuspended in pre‑warmed RPMI 1640 medium (Gibco) supplemented with 10% FBS. Approximately 5 × 10^5^ cells were seeded into 12‑well plates and allowed to adhere for 2 h at 37°C under 5% CO_2_. Non‑adherent cells were removed by washing, and the adhered macrophages were cultured overnight. Prior to infection, log‑phase *H. pylori* (10^9^ CFU in PBS) were opsonized by incubation with immune serum or PBS control serum collected from mice at 8 weeks post‑immunization for 1.5 h at 37°C. Macrophages were then co‑incubated with bacteria at a ratio of 10^9^ bacteria per well for 30 min. After infection, cells were washed with PBS and incubated with Amoxicillin (100 µg mL^−^^1^) in RPMI for 30 min to kill extracellular bacteria. Following gentamicin treatment, macrophages were washed and further incubated in antibiotic‑free RPMI for 0 or 60 min. Cells were subsequently lysed with 0.1% Triton X‑100, and the number of internalized *H. pylori* was quantified by plating serial dilutions on Columbia blood agar and counting CFU.

### 2.12 Urease Test

Mice were challenged with 109 CFU of *H. pylori* SS1. Two weeks later, the mice were euthanized and their gastric tissues were collected for urease detection. The gastric mucosal tissues of each group of mice were aseptically isolated and placed in 500 µL of 0.8% NaCl solution, and thoroughly ground at low temperature. A 100 µL aliquot of homogenate was added to 3 mL of urea broth (containing phenol red) and incubated at 37°C for 4 h. A homogenate from PBS‐treated mice served as the negative control. The OD value at a wavelength of 550 nm was determined using a spectrophotometer to represent its gastric urease activity.

### 2.13 Bacterial Load Determination

Similarly, two weeks after the challenge, all the gastric tissues of the mice were collected for bacterial quantification to evaluate the protective effect induced by immunity. Half of the stomach of each mouse was removed and rinsed with PBS. The tissue was weighed, placed in a pre‐weighed tube containing sterile BHI broth, and homogenized. Transfer it to a sterile homogenizer for thorough grinding. After grinding, dilute it at 1:10, 1:100 and 1:1000 respectively. Take 100 µL of the original solution and the diluted solution after grinding and spread them respectively on the agar plates containing vancomycin and FBS for brain heart extract. Incubate in a three‐gas incubator at 37°C for 6–7 days. Colonies were identified as *H. pylori* based on urease reaction, oxidase reaction and wet patch morphology. The load of *H. pylori* was calculated based on the dilution ratio, gastric tissue weight and the number of colonies on the plate.

### 2.14 Histopathology

Following *H. pylori* challenge, gastric tissues were harvested after two weeks for histopathological evaluation based on an established scoring system (Ghasemi et al. 2022). In brief, stomach samples were fixed in 10% neutral buffered formalin, processed routinely, embedded in paraffin, and sectioned. Tissue sections were stained with hematoxylin and eosin. A pathologist blinded to the experimental groups examined both the glandular and squamous regions of the stomach. Lesions were scored (0–6) for the following parameters: mucosal inflammation (severity and type), submucosal inflammation (severity and type), mucosal ulceration, and hyperkeratosis in the squamous epithelium.

### 2.15 Immunohistochemistry and Immunofluorescence

Gastric tissue samples were fixed in 10% neutral buffered formalin, embedded in paraffin, and sectioned at a thickness of 4 µm. For immunohistochemical staining, sections were deparaffinized, rehydrated, and endogenous peroxidase activity was quenched with 3% H_2_O_2_ in methanol. After blocking with 5% serum, sections were incubated overnight at 4°C with primary antibodies against IL‐17A or IFN‐γ. Following PBS washes, sections were incubated with horseradish peroxidase‐conjugated secondary antibodies for 1 hour at room temperature. Antigen detection was performed using a diaminobenzidine (DAB) substrate kit, and sections were counterstained with hematoxylin.

For immunofluorescence staining, a similar tissue preparation and blocking procedure was followed. Sections were incubated overnight at 4°C with an anti‐CD45 primary antibody. After washing, antigen detection was achieved using a fluorophore‐conjugated secondary antibody. Cell nuclei were counterstained with DAPI, and slides were mounted with an anti‐fade medium. All stained sections were visualized and imaged using a fluorescence microscope.

### 2.16 Flow Cytometer Analysis

Two weeks post *H. pylori* challenge, spleen lymphocytes were isolated using a mouse spleen lymphocyte isolation solution kit (Solarbio), single‐cell suspensions were then plated in 12‐well plates and stimulated with 6 µg/mL UreB protein or OMPs (endotoxin <100 EU/mg) in RPMI‐1640 medium supplemented with 100 IU/mL penicillin‐streptomycin and 10% FBS (Gibco), followed by incubation at 37°C under 5% CO_2_ for 24 h. During the final 6 h of incubation, protein transport inhibitors brefeldin A (2 µL/mL; BD Biosciences) and monensin (1.4 µL/mL; BD Biosciences) were added to the cells. After centrifugation and supernatant removal, 2 µL of Fc receptor blocker (BioLegend) and 5 µL of pre‐mixed True‐Stain monocyte blocker (BioLegend) were added to the cell pellet and incubated for 10 min. Subsequently, 2 µL of anti‐CD4 antibody (BioLegend) was introduced, followed by 25‐min incubation in the dark. Following PBS washes, cells were resuspended in 1 mL of diluted Zombie Aqua viability dye (BioLegend), incubated for 10 min, and centrifuged. Cells were then fixed, permeabilized, and washed according to the manufacturer's protocol (BD Fixation/Permeabilization Kit), with gentle resuspension after each centrifugation step.

For intracellular staining, a pre‐mixed cocktail containing Brilliant Stain Buffer Plus (BD Biosciences), True‐Stain monocyte blocker (BioLegend), anti‐CD154 (0.5 µg), anti‐IFN‐γ (1 µg), and anti‐IL‐17A (0.25 µg) antibodies (all from BioLegend) was prepared in PBS at a final volume of 100 µL per 1×10^6^ cells. This cocktail was thoroughly mixed with the cell pellet and incubated for 30 min at 4°C in the dark. Finally, cells were washed with BD Perm/Wash buffer and PBS, resuspended in 0.2 mL PBS, and acquired on a BD FACSVerse flow cytometer for data analysis.

### 2.17 Statistical Analysis

All statistical analyses were performed using GraphPad Prism 9.5.1 software. Data were analyzed by one‐way or two‐way analysis of variance (ANOVA) followed by Tukey's multiple comparisons test. Data were expressed as means ± standard deviation (SD). *P* < 0.05 was considered statistically significant (*P < 0.05; **P < 0.01; ***P < 0.001).

## 3 Results

### 3.1 Formulation and Characterization of Cytokine‐Loaded ENAP

Host‐mimicking LPS structures in *H. pylori* enable evasion of host immune clearance, promoting colonization. To counter this immune evasion, increase the immunogenicity of outer membrane vesicles (OMVs), and simultaneously reduce endotoxicity, we previously engineered *H. pylori* LPS by deleting *futB*, *lpxE*, and *lpxF* (Liu et al. 2025). Here, using LPS‐modified recombinant outer membrane vesicles (rOMVs) from *H. pylori*, we developed a customizable, cytokine‐presenting ENAP and assessed its adjuvant performance.

**FIGURE 1:**
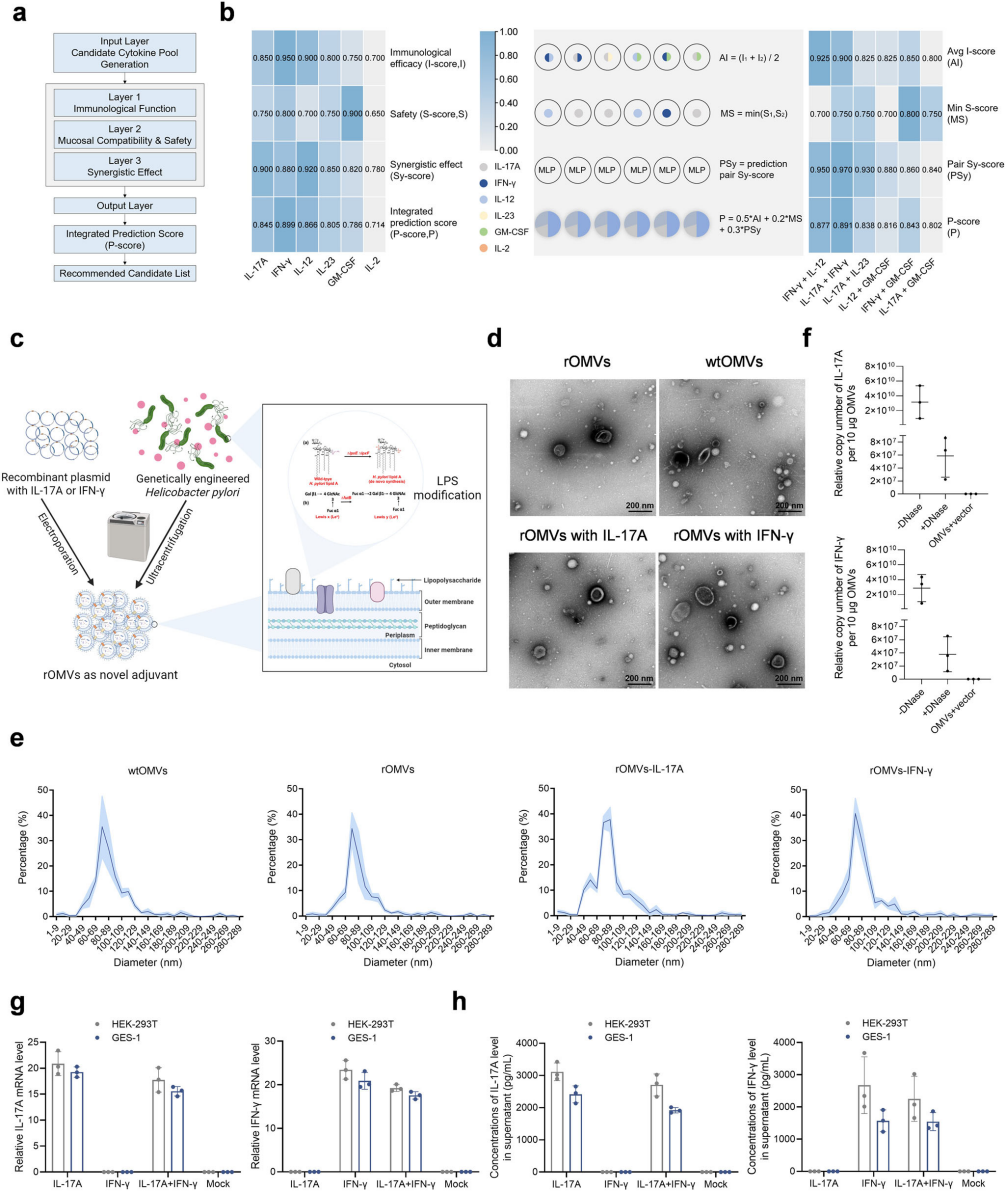
Design and characterization of the ENAP. (a) Screening workflow for AI‐guided selection of cytokines to be delivered by genetically engineered *H. pylori* OMVs for use as vaccine adjuvants against *H. pylori*. (b) Scores for the six top‐ranking individual cytokines and their combinations, evaluated by an AI‐driven framework based on mucosal immunity, Th1/Th17 responses, IgA induction, and safety. For cytokine combinations, the following weights were assigned to calculate the composite score: immunological efficacy (0.5), safety profile (0.2), and synergistic effect (0.3). (c) Schematic illustration of the construction of cytokine‐loaded ENAP: rOMVs encapsulating the IL‐17A eukaryotic expression plasmid and rOMVs encapsulating the IFN‐γ eukaryotic expression plasmid. (d) Representative transmission electron microscope (TEM) images of wild‐type OMVs (wtOMVs), recombinant OMVs (rOMVs), rOMVs loaded with IL‐17A plasmid, and rOMVs loaded with IFN‐γ plasmid. Scale bars, 200 nm. (e) Hydrodynamic particle size distribution of the prepared vesicles as determined by dynamic light scattering (DLS). (f) Quantification of encapsulated plasmid DNA by quantitative PCR. DNase I treatment was used to degrade unencapsulated (superficially attached) plasmid, with untreated samples as controls. (g and h) IL‐17A and IFN‐γ expression levels in HEK‐293T or GES‐1 cells transfected with OMV‐delivered cytokine‐encoding plasmids, as determined by qRT‐PCR (g) and ELISA (h). Data are presented as means ± SD from three independent experiments. Ordinary one‐way analysis of variance (ANOVA) was performed for all comparisons (*P < 0.05, **P < 0.01, ***P < 0.001).

The rapid advancement of AI has opened new avenues for its application in biomedical research. Applying AI to vaccine design holds great promise for significantly improving the precision and efficacy of the development process. Here, based on the desired characteristics of an adjuvant for *H. pylori* vaccines, we employed an AI‐based approach to screen for and identify promising cytokine candidates for delivery via genetically engineered *H. pylori* OMVs (Figure 1a). The selected cytokines were subsequently evaluated and scored for both their individual adjuvant potential and their synergistic effects (Figure 1b). To further potentiate adjuvanticity, we loaded eukaryotic expression plasmids encoding IL‐17A and IFN‐γ—cytokines that drive Th1 and Th17 responses—into OMVs via electroporation (Figure 1c), thereby increasing their capacity to promote clearance of infection. We evaluated the impact of LPS modification and plasmid loading on OMV properties by transmission electron microscopy (TEM), which revealed that LPS modification did not discernibly alter spherical morphology; OMVs remained uniformly dispersed spheres irrespective of plasmid loading (Figure 1d). Consistent with these observations, OMVs exhibited comparable diameters regardless of LPS modification or plasmid payload (Figure 1e). Quantitative PCR of preparations with or without DNase treatment (±DNase) quantified encapsulated plasmids and showed that a substantial fraction of the eukaryotic expression plasmids resided inside OMVs (Figure 1f). When plasmid‐loaded rOMVs were co‐incubated with HEK‐293T and GES‐1 cells, quantitative reverse transcription polymerase chain reaction (qRT‐PCR) and ELISA revealed substantially higher levels of IFN‐γ and IL‐17A than those induced by rOMVs loaded with empty plasmid in either cell line (Figure 1g,h). These data establish that OMVs efficiently deliver IFN‐γ or IL‐17A expression plasmids to eukaryotic cells, resulting in robust cytokine expression.

### 3.2 ENAP Elicits Sustained Systemic and Mucosal Immunity against Heterologous Antigens

**FIGURE 2:**
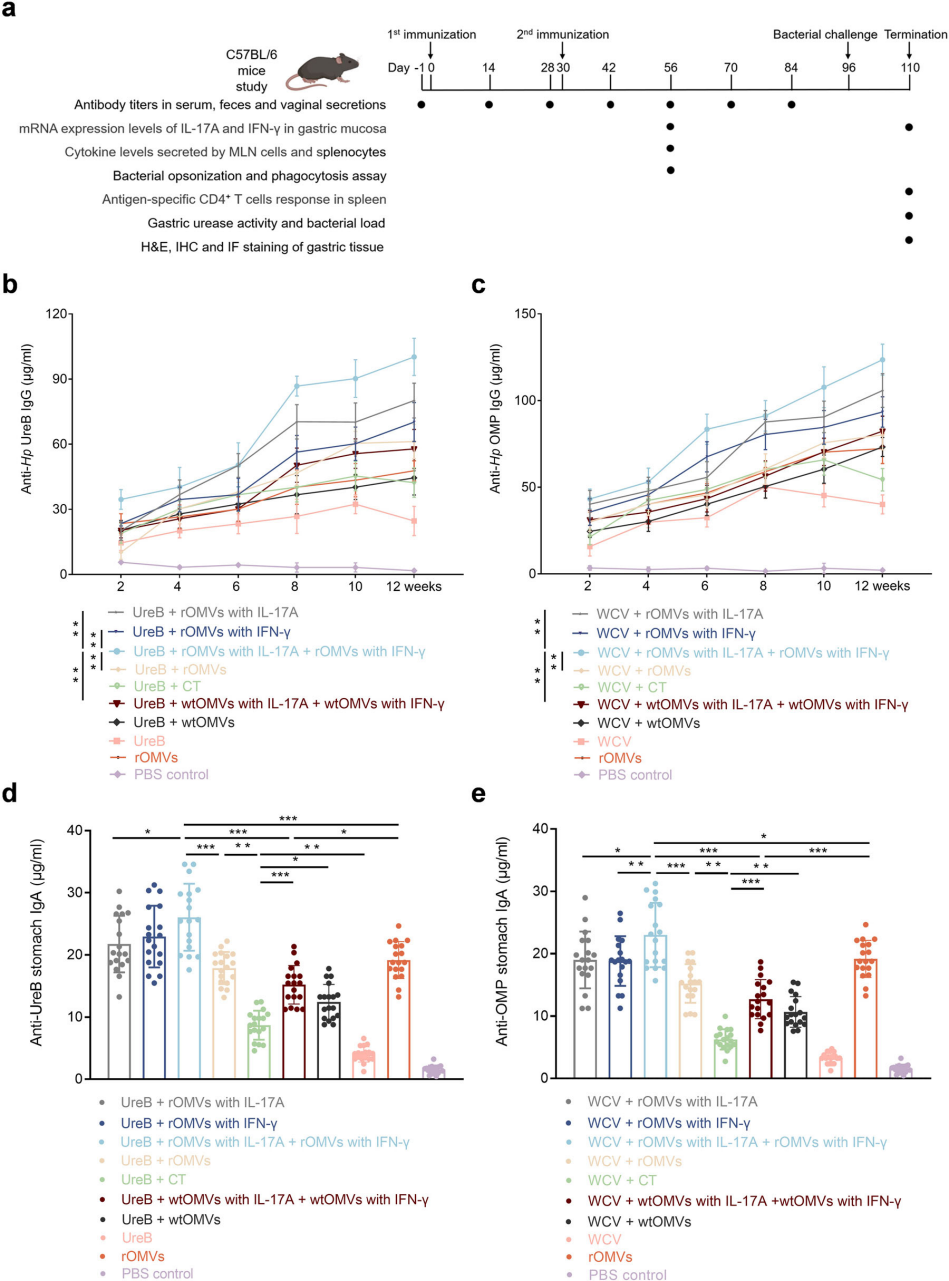
Cytokine‐loaded ENAP elicits sustained systemic and mucosal immunity against heterologous antigens. (a) Vaccination schedule and sample collection timeline. Serum, fecal, and vaginal wash samples were collected at the indicated time points. Antigen‐specific IgG antibody titers against UreB and OMP in serum were determined by ELISA. Wild‐type *H. pylori* outer membrane vesicles (wtOMVs) and CT were used as control adjuvants; PBS was used as a negative control. (b and c) Longitudinal monitoring of serum anti‐UreB (b) and anti‐OMP (c) IgG titers over 12 weeks after immunization with UreB or WCV as antigens and OMVs as an adjuvant platform, measured by ELISA. *n* = 18 mice (9 per group from two independent experiments); data were pooled for statistical analysis. Statistical comparisons shown are for the endpoint (12‐week) titers. (d and e) Gastric mucosal anti‐UreB stomach IgA (d) and anti‐OMP stomach IgA (e) levels measured by ELISA in stomach homogenates from euthanized mice at week 8 post‐immunization, using UreB or WCV as the coating antigen. Data are pooled from two independent experiments (*n* = 9 mice per group). Data are presented as means ± SD. Ordinary one‐way ANOVA was performed for all comparisons (*P < 0.05, **P < 0.01, ***P < 0.001).

To determine whether ENAP loaded with IL‐17A or IFN‐γ eukaryotic expression plasmids (rOMVs with IL‐17A or IFN‐γ) enhance immunity across distinct antigen formats, we systematically compared durable systemic and mucosal responses in mice using formulations containing either a recombinant UreB subunit antigen or an inactivated whole‐cell antigen (killed *H. pylori*). Following an immunization schedule adapted from prior work (Liu et al. 2023), six‐week‐old C57BL/6 mice (*n* = 9) were acclimated for 1 week, then orally immunized (200 µg antigen + 10 µg adjuvant) on day 0 and boosted on day 30. Serum, fecal, and vaginal wash samples were collected from one day before immunization through day 84; gastric mucosa, mesenteric lymph nodes, and splenocytes on day 56; and spleen and gastric mucosa were harvested 2 weeks post‐challenge (Figure 2a).

First, cytokine‐loaded ENAP vaccination significantly increased the infiltration of CD45^+^ immune cells in the gastric mucosa (FigureS1a–d). This provides direct histological evidence that ENAP effectively induces and activates local mucosal immunity in situ. Then, we quantified the kinetics of antigen‐specific total IgG and IgA titers by ELISA. A 1:1 combination of IL‐17A‐ and IFN‐γ‐loaded rOMVs, when used as an adjuvant, maintained higher antigen‐specific IgG titers at all time points regardless of whether it was combined with the UreB antigen or the WCV antigen. The endpoint titers persisted 12 weeks post‐immunization, significantly exceeding those induced by CT or wild‐type OMVs (Figure 2b,c). Moreover, the rOMVs‐plasmid adjuvant markedly increased week‐8 gastric mucosal IgA titers (Figure 2d,e) and sustained antigen‐specific responses in vaginal secretions (Figure S2a,b), serum (Figure S2c,d), and fecal IgA (Figure S2e,f), indicating stronger and more durable mucosal immunity.  Notably, the engineered OMVs produced tissue‐specific kinetic patterns: vaginal SIgA continued to increase at week 8 post‐immunization, whereas fecal and serum IgA—though higher than with CT—gradually declined. Moreover, IL‐17A plasmid loaded OMVs may upregulate CCL28 secretion, establishing a positive feedback loop that further increases plasma cell residence time (Sun et al. 2013). In the meantime, analysis of the antibody responses against the OMV adjuvant itself revealed that mice immunized with the WCV antigen generated significantly higher anti‐OMV antibody titers compared to those receiving the UreB antigen (Figure S3a–d). This differential response suggests that the native WCV preparation and the OMVs share a broader repertoire of common antigens, and that these shared components likely play a key role in driving the observed humoral and mucosal immunity. These findings highlight a promising strategy for future vaccine optimization, whereby the identification and selective inclusion of these immunodominant shared antigens from the OMV membrane could guide the rational design of more effective multi‐epitope vaccines.

### 3.3 ENAP Delivering Cytokines as Adjuvants Elicited IL‐17A and IFN‐γ Secretion

**FIGURE 3:**
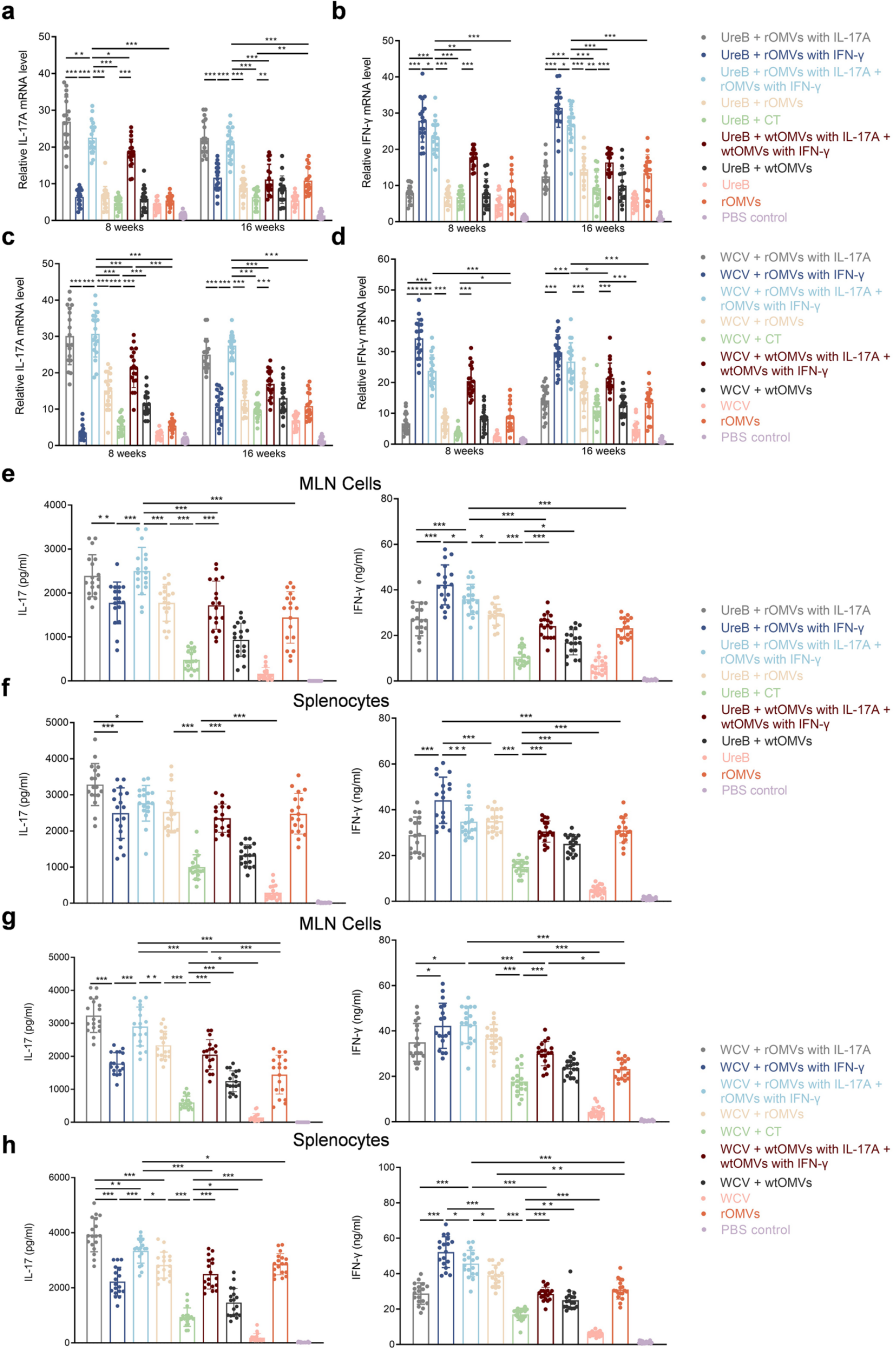
ENAP induces mucosal and systemic IL‐17A and IFN‐γ responses. (a–d) mRNA levels of IL‐17A (a, c) and IFN‐γ (b, d) in the gastric mucosa of immunized mice at the indicated time points (8 weeks and 16 weeks post‐immunization; challenge was performed 2 weeks prior to the 8‐week measurement). a and b: UreB antigen; c and d: WCV antigen. Data are pooled from two independent experiments (*n* = 9 mice per group). (e–h) IL‐17 and IFN‐γ production in supernatants from mesenteric lymph node (MLN) cells and splenocytes restimulated with UreB (e, f) or OMP (g, h) for 24 h at week 8, quantified by ELISA. Mice were immunized with the indicated antigen (UreB or WCV) combined with the specified adjuvant (ENAP, wtOMVs, or CT). Data are pooled from two independent experiments (*n* = 9 mice per group) and presented as means ± SD. Statistical analysis was performed by ordinary two‐way ANOVA (a–d) or ordinary one‐way ANOVA (e–h) (*P < 0.05, **P < 0.01, ***P < 0.001).

To define the overall immune responses in mice induced by ENAP carrying IL‐17A or IFN‐γ combined with UreB or WCV, we quantified cytokine mRNA by qRT‐PCR to assess the durable adjuvant efficacy at both 8 and 16 weeks post‐immunization. At week 8 post‐immunization (and two weeks after *H. pylori* challenge), across all antigen pairings, the cytokine‐loaded ENAP group (rOMVs with IL‐17A + rOMVs with IFN‐γ) showed significantly higher IL‐17A and IFN‐γ transcripts than the unloaded group. Notably, this enhanced cytokine expression was sustained and remained significantly elevated at 16 weeks post‐immunization, further confirming the durable adjuvant effect of the cytokine‐loaded ENAP platform (Figure 3a–d). This sustained elevation indicates that the increased cytokine levels are primarily attributable to delivery of eukaryotic expression plasmids rather than an intrinsic adjuvant effect of OMVs, and further suggests that cytokine plasmids carried by engineered rOMVs are key contributors to durable immune responses in mice.

Next, on day 56 post‐prime, we isolated mesenteric lymph node cells and splenocytes and measured cytokine secretion by ELISA after OMP or UreB stimulation; consistently, the plasmid‐loaded rOMV groups (rOMVs with IL‐17A + rOMVs with IFN‐γ) secreted higher levels of IL‐17A and IFN‐γ than the unloaded groups, irrespective of pairing with UreB or WCV (Figure 3e–h). Together, these findings demonstrate that genetically engineered rOMVs delivering functional cytokine plasmids concurrently amplify mixed Th1/Th17 responses in mucosal lymphoid tissue (MLN) and the spleen, providing valuable insights for vaccine design.

### 3.4 ENAP Enhances Protective Immunity Against *H. Pylori* Infection

**FIGURE 4:**
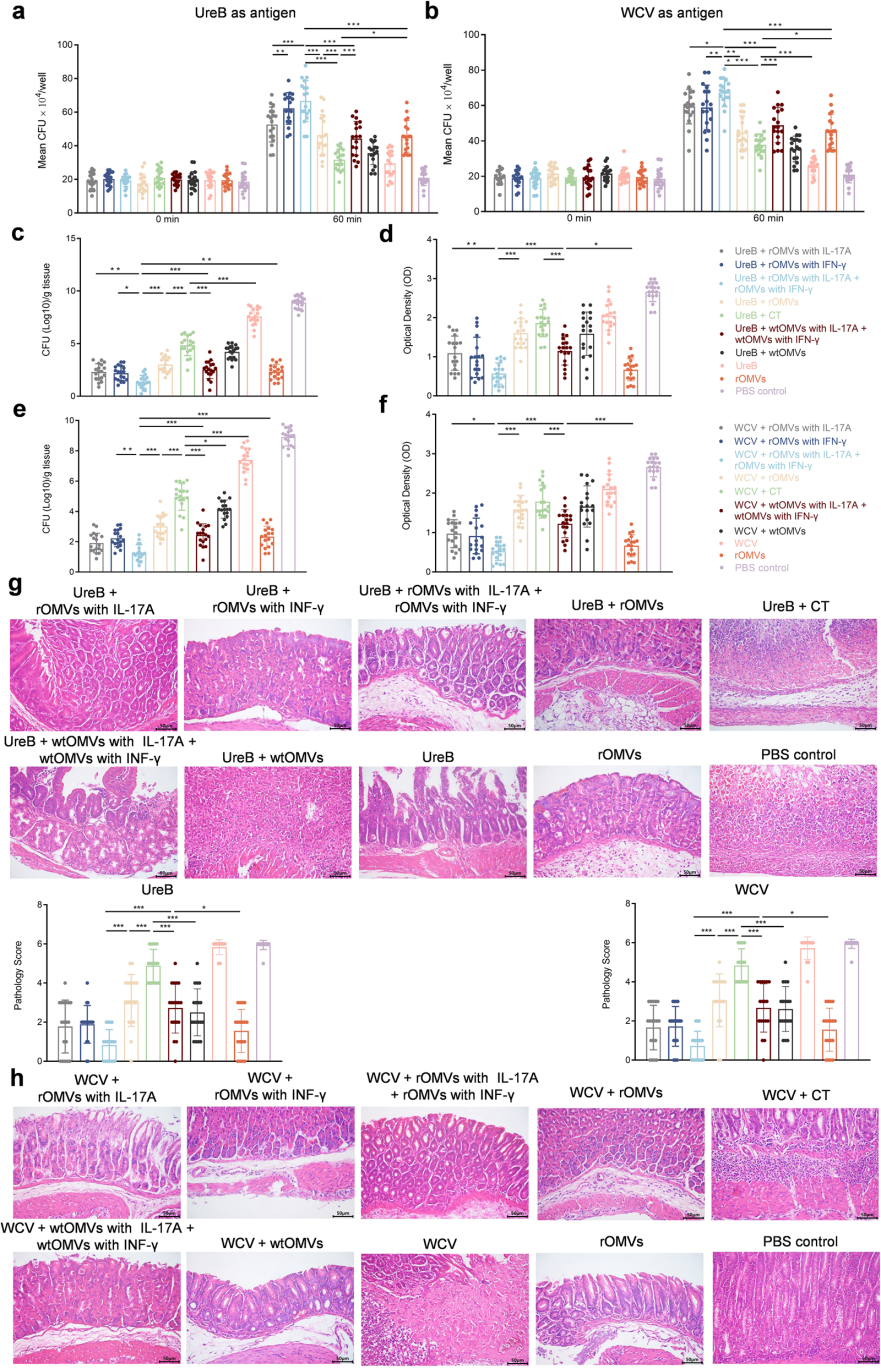
ENAP enhances protective immunity against *H. pylori* infection. (a and b) Opsonophagocytic activity of sera from mice immunised with OMV‐delivered cytokines in vitro. (c and e) Bacterial load in stomachs of mice collected two weeks post‐challenge (day 110), determined by quantifying *H. pylori* colony‐forming units (CFU) after plating stomach homogenates. (d and f) Urease activity measured in gastric tissue homogenates two weeks post‐challenge (day 110). (g and h) Representative hematoxylin and eosin staining images (left) and corresponding pathology scores (right) of gastric tissues from mice immunized with UreB (g) or WCV (h) combined with the indicated adjuvants. The pathology of gastric mucosa was scored on a scale of 0–6 based on the combined assessment of inflammatory infiltration, epithelial damage, and other histological alterations. Scale bars, 50 µm. Data are pooled from two independent experiments (*n* = 9 mice per group) and presented as means ± SD. Ordinary one‐way ANOVA was performed for all comparisons (*P < 0.05, **P < 0.01, ***P < 0.001).

Having established that ENAP elicits durable, broad antibody responses, we systematically evaluated the protective efficacy of cytokine‐loaded OMVs as adjuvants against *H. pylori*. First of all, we performed an ex vivo macrophage model to examine the opsonophagocytic activity of immune serum. We observed that, regardless of the antigen paired with the cytokine‑loaded OMVs, bacterial uptake was significantly enhanced in the presence of immune serum from vaccinated mice (Figure 4a,b). These results indicate that immunization with the ENAP platform promotes the activation and phagocytic activity of macrophages against *H. pylori*, further supporting the functional potency of the vaccine‑induced humoral response. Then, using a standardized protection assay, we confirmed that cytokine‐loaded ENAP (rOMVs with IL‐17A + rOMVs with IFN‐γ) significantly enhances protection. After two immunizations, mice were challenged on day 96 with 10^9^ CFU of *H. pylori* strain SS1 and monitored for two weeks. Gastric mucosae were then collected for protection readouts. Quantification of gastric bacterial burden revealed that, regardless of antigen pairing, mice receiving IL‐17A/IFN‐γ‐loaded rOMVs had the lowest gastric *H. pylori* burden, which was significantly lower than that in the dual‐cytokine wtOMVs group (Figure 4c,e). Importantly, the dual‐cytokine regimen (rOMVs with IL‐17A + rOMVs with IFN‐γ) achieved greater reductions in bacterial loads than single‐cytokine regimens (rOMVs with IL‐17A or IFN‐γ), consistent with synergy. Correspondingly, urease activity assays showed similar decreasing trends among the OMV adjuvant treatment groups (Figure 4d,f). This additive protection may reflect cooperative Th1/Th17 responses: IL‐17A enhances mucosal neutrophil recruitment to clear early colonizers, whereas IFN‐γ activates macrophage‐mediated killing of residual intracellular pathogens, jointly promoting immune clearance. Gastric histopathology corroborated these findings. Hematoxylin and eosin (H&E) staining revealed reduced inflammatory infiltration, preserved glandular architecture, and minimal mucosal loss in mice treated with dual‐cytokine‐loaded ENAP. In contrast, the PBS control group displayed pronounced inflammatory infiltrates and mucosal injury after challenge (Figure 4 g,h). Collectively, these findings indicate that cytokine‐loaded ENAP enhances protective immunity against *H. pylori* infection.

### 3.5 ENAP Promotes Balanced Th1, Th2, and Th17 Immune Responses in Mice

**FIGURE 5:**
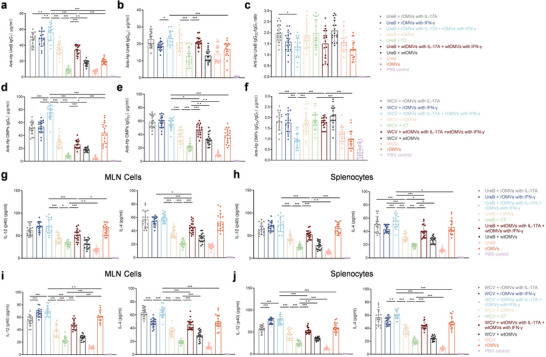
ENAP enhances Th1, Th2, and Th17 immune responses. (a–f) Serum anti‐UreB IgG1 (a), IgG2c (b), and anti‐OMP IgG1 (d), IgG2c (e) levels at week 8 (day 56), measured by ELISA. The IgG2c/IgG1 ratios for anti‐UreB (c) and anti‐OMP (f) are shown. (g to j) Production of IL‐12(p40) (g, i) and IL‐4 (h, j) measured by ELISA in supernatants from MLN cells and splenocytes isolated 8 weeks post‐immunization, upon restimulation with UreB (g, h) or OMP (i, j). Mice were immunized with the indicated antigen combined with ENAP or CT adjuvant. Data are pooled from two independent experiments (*n* = 9 mice per group) and presented as means ± SD. Ordinary one‐way ANOVA was performed for all comparisons (*P < 0.05, **P < 0.01, ***P < 0.001).

In mice, IgG1 and IgG2c serve as canonical markers of Th2‐ and Th1‐skewed immune responses, respectively; accordingly, the IgG2c/IgG1 ratio is positively correlated with Th1 and inversely correlated with Th2 immunity (Steinbuck et al. 2021). To define the immune correlates of protection mediated by cytokine‐loaded OMV adjuvants, we comprehensively profiled humoral and cellular responses elicited by the recombinant vaccines. Serum isotype profiling showed that, relative to other groups, rOMVs loaded with IL‐17A and IFN‐γ increased both IgG1 and IgG2c, resulting in a balanced Th1/Th2 response (Figure 5a–f). At the cellular level, the combination of rOMVs with IL‐17A and rOMVs with IFN‐γ concomitantly increased secretion of IL‐4 (Th2), IL‐12p70 (Th1), and IL‐6 in both MLN cells and splenocytes, irrespective of antigen type (WCV or UreB) (Figure 5g–j). Although dual‐cytokine rOMVs immunization increased IL‐6 (Figure S2a–d), no OMV‐immunized mice showed abnormal behaviour, marked weight changes (Figure S3a,b), altered food intake, or other adverse events; animals remained healthy throughout the study, indicating that low‐dose, engineered OMVs used as adjuvants do not elicit overt toxicity and induce only transient, physiologically contained inflammation.

To further validate these findings, we assessed antigen‐specific T‐cell activation by flow cytometry in spleens two weeks after *H. pylori* challenge: splenocytes from each group were stimulated with UreB or OMPs, and the frequencies of CD4^+^CD154^+^, CD154^+^IL‐17A^+^, and CD154^+^IFN‐γ^+^ T cells were quantified. Consistent with these results, mice immunized with cytokine‐loaded rOMVs (rOMVs with IL‐17A + rOMVs with IFN‐γ) displayed higher activation of CD4^+^CD154^+^, CD154^+^IL‐17A^+^, and CD154^+^IFN‐γ^+^ T cells, supporting Th1 and Th17 polarization (Figure S4a–f). Notably, high‐dose rOMVs alone also elicited elevated antigen‐specific T‐cell responses; we speculate that this may be related to the mechanism whereby high‐dose OMVs activate pre‐existing memory CD4^+^ T cells via antigen presentation through major histocompatibility complex (MHC) II molecules on dendritic cells (Kunzli and Masopust 2023, Racle et al. 2023). Furthermore, we performed immunohistochemical (IHC) staining for IL‐17A and IFN‐γ in the gastric mucosa of challenged mice to evaluate their roles in immune protection. The results demonstrated that following immunization with IL‐17A and IFN‐γ‐loaded rOMVs combined with antigen, both IL‐17A (Figure S5a,b) and IFN‐γ (Figure S6a,b) levels remained markedly elevated within the gastric mucosa. In sum, the ENAP‐based recombinant *H. pylori* vaccine robustly augments Th1/Th2/Th17 responses in mice, an enhancement that likely constitutes the foundation for the observed protective immunity against *H. pylori* infection.

### 3.6 Validation of ENAP's Protective Efficacy in IL‐17A^−^/^−^ and IFN‐γ^−^/^−^ Murine Models

**FIGURE 6:**
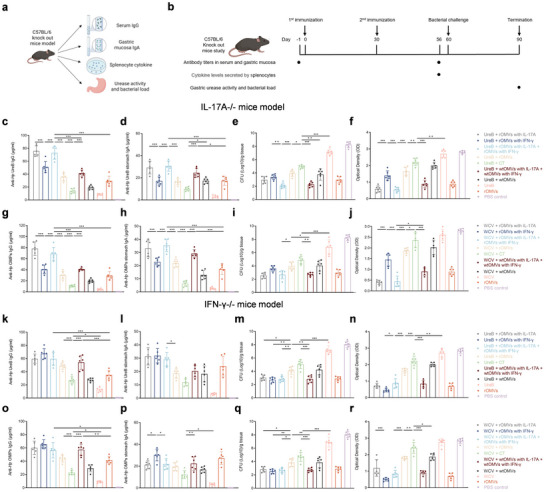
Validation of ENAP efficacy in IL‐17A^−^/^−^ and IFN‐γ^−^/^−^ murine models. (a and b) Vaccination and challenge schematics for IL‐17A^−^/^−^ and IFN‐γ^−^/^−^ mouse models. Serum and gastric mucosa were collected one day prior to the first immunization and 56 days post‐immunization for measurement of antigen‐specific IgG and IgA by ELISA. Splenocytes were isolated on day 56 for cytokine analysis. Gastric tissues were collected 30 days post‐challenge for bacterial load and urease activity assessment. wtOMVs and CT were used as control adjuvants; PBS served as a negative control. (c, g, k, o) Serum anti‐UreB IgG (c, k) and anti‐OMP IgG (g, o) titers in immunized IL‐17A^−^/^−^ (c, g) and IFN‐γ^−^/^−^ (k, o) mice, measured by ELISA. *n* = 6 mice per group. (d, h, l, p) Gastric mucosal anti‐UreB IgA (d, l) and anti‐OMP IgA (h, p) levels in IL‐17A^−^/^−^ (d, h) and IFN‐γ^−^/^−^ (l, p) mice, measured by ELISA at week 8. *n* = 6. (e, i, m, q) Bacterial load (CFU) in stomachs of IL‐17A^−^/^−^ (e, i) and IFN‐γ^−^/^−^ (m, q) mice one month post‐challenge (day 90). *n* = 6. (f, j, n, r) Urease activity in gastric homogenates from IL‐17A^−^/^−^ (f, j) and IFN‐γ^−^/^−^ (n, r) mice one month post‐challenge (day 90). Data are presented as means ± SD (*n* = 6 mice per group). Ordinary one‐way ANOVA was performed for all comparisons (*P < 0.05, **P < 0.01, ***P < 0.001).

To define the mechanism by which rOMV adjuvants loaded with IL‐17A and IFN‐γ confer protection, we performed loss‐of‐function validation in IL‐17A^−^/^−^ and IFN‐γ^−^/^−^ knockout mice. Six‐week‐old IL‐17A^−^/^−^ and IFN‐γ^−^/^−^ mice were acclimated for one week and then orally gavaged with vaccines containing 200 µg antigen and 10 µg adjuvant. Animals were primed on day 0 and boosted on day 30. Serum was collected one day before immunization and on day 56. Gastric mucosae and splenocytes were harvested on day 56. Gastric mucosae were collected two weeks after challenge for downstream analyses (Figure 6a,b). In IL‐17A^−^/^−^ mice, rOMVs carrying the IL‐17A expression plasmid (rOMVs with IL‐17A) increased antigen‐specific IgG and IgA titers relative to unloaded rOMVs (Figure 6c,d,g and h) and conferred stronger protection against *H. pylori*, as evidenced by lower bacterial burden and urease activity (Figure 6e,f,i and j). Likewise, in IFN‐γ^−^/^−^ mice, rOMVs carrying the IFN‐γ expression plasmid (rOMVs with IFN‐γ) significantly increased antigen‐specific IgG and IgA titers relative to unloaded controls and enhanced protection against *H. pylori*, resulting in comparable levels of immunoprotection (Figure 6k–r). Furthermore, in IL‐17A^−^/^−^ mice immunized with cytokine‐loaded OMVs plus either UreB or WCV antigen, the resulting anti‐OMV IgA antibody titers were significantly lower (Figure S9b,d). This reduction is consistent with the impaired immunity observed when UreB or OMPs themselves were used as the coating antigens in immunoassays. Similarly, analysis of anti‐OMV antibody titers revealed that mice immunized with the WCV formulation mounted significantly stronger OMVs‐specific responses than those receiving the UreB antigen (Figure S9a,c,e–h), indicating that the broader antigenic overlap between WCV and the OMVs backbone enhances the overall immunogenicity of the vaccine platform.

Together, these data indicate that the protective activity of cytokine‐loaded rOMVs depends on the function of the delivered cytokines; although rOMVs retain innate TLR ligand‐mediated adjuvanticity, they do not compensate for the immune deficits caused by loss of IL‐17A or IFN‐γ. These findings support two design principles: (i) precise targeting of carrier function—rOMVs act as a targeted‐delivery platform for cytokines, and their adjuvant effect must be coupled to targeted delivery to achieve maximal protection; and (ii) the generalizability of the rOMVs platform—genetic‐deficiency models demonstrate the functional necessity of cytokine cargo, providing a mechanistic basis for deploying alternative cytokine combinations in future vaccine designs.

### 3.7 Elucidating ENAP Mechanisms via Cytokine Analysis in IL‐17A^−^/^−^ and IFN‐γ^−^/^−^ Mice

**FIGURE 7:**
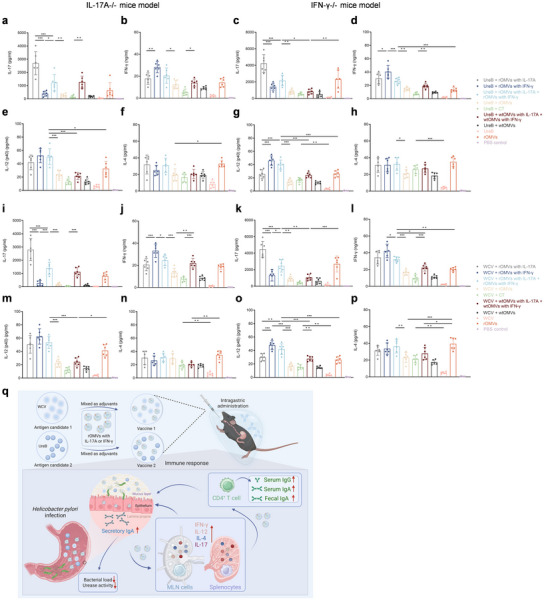
Cytokine profiling in IL‐17A^−^/^−^ and IFN‐γ^−^/^−^ mice delineates immune mechanisms of ENAP. (a–p) Production of IL‐17 (a, c, i, k), IFN‐γ (b, d, j, l), IL‐12(p40) (e, g, m, o), and IL‐4 (f, h, n, p) measured by ELISA in supernatants from splenocytes isolated from immunized IL‐17A^−^/^−^ (a–h, q) and IFN‐γ^−^/^−^ (i–p, r) mice 8 weeks post‐immunization, after restimulation with UreB (a to h) or OMP (i–p). Mice were immunized with the indicated antigen (UreB or WCV) combined with ENAP or CT adjuvant. *n* = 6 mice per group. (q) Proposed mechanism: the *H. pylori* recombinant vaccine, composed of cytokine‐presenting ENAP combined with UreB or WCV antigen, elicits durable humoral, mucosal, Th1‐, Th2‐, and Th17‐type cellular immune responses upon immunization, ultimately protecting mice against *H. pylori* infection. Data are presented as means ± SD (*n* = 6 mice per group). Ordinary one‐way ANOVA was performed for all comparisons (*P < 0.05, **P < 0.01, ***P < 0.001).

Given the limited yield of mesenteric lymph node cells in IL‐17A^−^/^−^ and IFN‐γ^−^/^−^ mice, we focused on splenocytes and measured by ELISA the secretion of IL‐17A, IFN‐γ, IL‐12p40, and IL‐4 at week 8 post‐immunization after restimulation with OMPs or UreB, thus defining the cytokine‐mediated mechanisms of action of cytokine‐loaded rOMV adjuvants in knockout mice. Across genetic backgrounds and antigen pairings, rOMVs with IL‐17A increased IL‐17A (Th17) secretion (Figure 7a,c,i and k). Similarly, rOMVs with IFN‐γ increased secretion of IFN‐γ (Figure 7b,d,j and l) and IL‐12p40 (Figure 7e,g,m and o) (Th1). By contrast, IL‐4 (Th2) secretion did not differ between single‐ and dual‐cytokine rOMVs groups (Figure 7f,h,n and p). Notably, the dual‐cytokine regimen (rOMVs with IL‐17A + rOMVs with IFN‐γ) achieved greater activation, consistent with synergy, across Th1 (IL‐12(p40)/IFN‐γ), Th2 (IL‐4), and Th17 (IL‐17A) while maintaining a balanced profile. This immune profile, characterized by dominant Th1–Th17 and maintained Th2 activity, may mitigate the pathological risks of overactivation of a single Th subset (Th17‐mediated inflammatory damage or Th2‐associated fibrosis) and supports the basis for safe, durable immune memory. These data further support the design logic and potent immunogenicity of our cytokine‐loaded ENAP.

In summary, coordinated activation of the mucosal barrier, together with durable immune memory, is pivotal for achieving effective immunity against *H. pylori*. We developed a customizable cytokine‐presenting ENAP, enabling stable, targeted, and low‐toxicity cytokine delivery. Co‐immunization of UreB or WCV with this adjuvant markedly augmented mucosal responses and Th1/Th17 cellular immunity, thereby limiting *H. pylori* colonization. Cooperation between cytokine‐armed rOMVs and antigen elicited pathogen‐specific adaptive responses and substantially improved protective efficacy in the challenge model (Figure 7q). This adjuvant system, integrating vector delivery with cytokine‐cascade amplification, offers an innovative strategy against *H. pylori*. Furthermore, due to its modular plasmid design, this platform can be readily extended to develop combination vaccines against other pathogens, underscoring broad translational potential.

## 4 Discussions

Nanomaterials offer enhanced antigen protection, enable immunomodulation and antigen delivery, and—owing to their potent immunostimulatory capacity—hold considerable promise for advanced adjuvant design. Numerous efforts are advancing the development of next‐generation nano‐adjuvants. For example, one recent study self‐assembled Mn^2+^ and CpG with epigallocatechin gallate (EGCG) to create a metal‐polyphenol network (MPN) adjuvant (MPN/CpG), which synergistically enhances STING signalling via Mn^2+^ and activates TLR9 via CpG, thereby eliciting potent antitumor T‐cell responses and suppressing tumour growth (Liu et al. 2024). Overall, co‐delivering antigens and molecular adjuvants with sophisticated nanomaterials is a highly promising strategy for efficient immune activation (Li et al. 2020). A key distinction, however, lies in the platform's origin and function: whereas most studies use synthetic nanoparticle formulations, our system is naturally derived, offering intrinsic biocompatibility and a high degree of amenability to precise functionalization. Its core innovation is multipronged immunomodulation via cytokine delivery, potentially affording a broader and more adaptable therapeutic window. We present, to our knowledge, a first‐in‐class, programmable‐cytokine ENAP that uses genetically engineered *H. pylori* rOMVs to achieve spatiotemporal control of cytokines. The platform encapsulates IL‐17A/IFN‐γ eukaryotic expression plasmids within rOMVs, leverages surface adhesins such as BabA for engagement with the gastric mucosal epithelium, and facilitates endosomal escape via membrane fusion, thereby limiting lysosomal degradation and enabling sustained plasmid release in the gastric mucosa. A central advantage is the creation of a microenvironment‐restricted cytokine niche, which may mitigate the risk of cytokine‐storm‐like toxicities associated with exogenous cytokine delivery. We found that cytokine‐loaded ENAP not only augments durable systemic immunity but also markedly potentiates mucosal immune responses. Importantly, we demonstrate that the heightened Th1/Th17 responses induced by the engineered nano‐vesicle adjuvant derive primarily from delivery of the eukaryotic expression plasmids rather than from intrinsic rOMVs adjuvanticity, a conclusion further corroborated in knockout mouse models. We envision these genetically engineered nano‐vesicles as efficient tools for personalized plasmid delivery, readily adaptable to other vaccines or gene therapy designs.

As a naturally derived class of nano‐vesicles, OMVs offer intrinsic advantages for small‐molecule delivery and vaccine development. Adhesins such as BabA and SabA displayed on *H. pylori* OMVs bind Lewis antigens with high specificity—enabling robust mucosal retention and potentiating immunologic effects (Doohan et al. 2021, Ansari and Yamaoka 2019). The OMVs’ lipid bilayer, together with dense LPS, forms a barrier that shields payloads from gastric proteases and nucleases. Additionally, OMVs‐associated urease buffers local pH across the gastric mucosa, regulating the mucus layer and facilitating deeper OMVs penetration (Turner et al. 2018, Jarzab et al. 2020). Beyond serving as efficient delivery vehicles, LPS‐engineered *H. pylori* OMVs represent a promising strategy to overcome immune evasion driven by molecular mimicry of host gastric epithelial blood‐group antigens, by exposing conserved antigenic epitopes and enhancing DC activation, thereby supporting effective immunity and reducing *H. pylori* colonization (Maldonado et al. 2016, Li et al. 2016). Induction of Th1 and Th17 responses by *H. pylori* vaccines has been repeatedly associated with enhanced protection and is widely considered a key correlate of protection (Liu et al. 2024, Ye et al. 2025, Xie et al. 2021). Accordingly, LPS‐modified OMVs strategies that amplify Th1/Th17 immunity represent a rational approach to potentiate macrophage‐mediated bactericidal activity and reinforce mucosal‐barrier defences. To address the efficacy‐safety trade‐off of mucosal adjuvants against *H. pylori*, we developed an ENAP in which rOMVs carry plasmids encoding IL‐17A or IFN‐γ and deliver them into eukaryotic cells for in situ expression. Across both the UreB subunit and an inactivated whole‐cell vaccine, ENAP increased serum IgG and gastric mucosal IgA, elicited dominant Th1/Th17 responses, and lowered gastric bacterial burden after challenge; the IL‐17A+IFN‐γ combination outperformed single‐cytokine formulations. These findings support ENAP as a safe, programmable strategy to enhance host immunity while enabling efficient cytokine delivery.

Several adjuvants have been used—or are in development—as components of *H. pylori* vaccines, including (i) bacterial toxin derivatives (e.g., *E. coli* LT and CT), (ii) classical inorganic adjuvants (e.g., aluminum salts), and (iii) biomaterial‐based adjuvants (e.g., chitosan). However, these adjuvants exhibit notable limitations, including safety concerns and insufficient immunogenicity (Pizza et al. 2001). Although recent studies have engineered less‐toxic mutants of LT and CT, or explored CpG or lactose as novel adjuvants, yielding encouraging results in animal models; however, none have achieved a balance between broad immune activation and pathogen‐specific protection (Holmgren et al. 2018, Stone et al. 2021, Crothers and Norton 2023, Qiao et al. 2022). Therefore, an ideal *H. pylori* vaccine adjuvant should: (i) efficiently induce mucosal and systemic immunity (especially Th1/Th17 polarization), (ii) elicit precise *H. pylori*‐specific clearance, and (iii) meet clinical‐grade safety standards. Notably, we found that cytokine‐loaded, LPS‐modified OMVs could meet these criteria. First, LPS modification substantially enhances safety. Second, *H. pylori*‐derived vesicles provide strong intrinsic immunogenicity. Finally, customizable cytokine presentation augments both mucosal and systemic immunity while improving cargo targeting, thereby enabling precise pathogen clearance.

In *H. pylori* vaccination, precisely balancing Th1, Th2, and Th17 responses is critical to achieving effective bacterial clearance while limiting immunopathology (Hitzler et al. 2011), with the key requirement being coordination of three functions: Th1 (intracellular bactericidal activity and clearance of deeply colonizing bacteria), Th17 (mucosal barrier defense and removal of surface colonizers), and Th2 (regulation of inflammation) (Ruterbusch et al. 2020). An optimized balance is characterized by a relatively stronger Th1 response, an appropriately calibrated Th17 response, and a tempered Th2 response. In this study, we formulated a 1:1 combination of rOMVs loaded with IL‐17A and IFN‐γ as an adjuvant. This formulation established a favourable Th1/Th17/Th2 balance, outperforming alum and single‐cytokine rOMVs controls, which exhibited skewed immunity. Moreover, by adjusting the proportion of OMVs carrying each plasmid, we can dynamically tune the Th1/Th17 axis to reduce the risk of immune deviation. This on‐demand, ratio‐adjustment strategy extends the platform to diverse vaccine designs, facilitating iterative optimization.

Although cytokine‐loaded ENAP drives efficient IL‐17A/IFN‐γ expression via plasmid delivery, the stability of electroporation‐based loading process and the inherently transient nature of plasmid expression may limit adjuvant durability and the formation of long‐lived immune memory. To improve loading stability, rigid sterol analogs can be included in the electroporation buffer to increase bilayer packing density and reduce pulse‐induced membrane defects (Miao et al. 2002). Tuning pulse parameters so that the applied field matches the rOMVs’ size distribution helps prevent electroosmotic overdrive and vesicle lysis. Additionally, to prolong cytokine action and improve mucosal‐barrier penetration, advanced molecular engineering can be integrated, such as transposon‐mediated genomic integration or CRISPR activation (CRISPRa) at endogenous loci (Chen et al. 2024, Yin et al. 2024, Liu et al. 2024). Further carrier optimization will be needed to overcome durability bottlenecks and advance the clinical translation of mucosal vaccines. In addition, several limitations remain. First, as noted above, only two candidate antigens were assessed; therefore, the compatibility of rOMVs with additional antigen classes requires further evaluation. Second, CT was the only control adjuvant used to assess the OMV‐based nano‐vesicle adjuvant; future work should include multiple, commonly used *H. pylori* adjuvants to rigorously benchmark this ENAP.

## 5 Conclusions

In summary, an rOMVs‐based ENAP that co‐delivers expression plasmids encoding IL‐17A and IFN‐γ enables efficient, targeted delivery, robustly induces mucosal immunity, and synergizes with co‐administered antigens, representing a promising next‐generation vaccine design strategy against *H. pylori* and other mucosal pathogens. Notably, this work demonstrates enhanced efficacy of the rOMVs adjuvant against *H. pylori* infection and establishes a modular vaccine‐carrier design framework; by tuning plasmid cargo ratios, the platform can be rapidly adapted to the specific immunologic requirements of diverse pathogens, providing a generalizable technical foundation for next‐generation nano‐adjuvants.

## Author Contributions

**Yinpan Shang**: conceptualization, methodology, investigation, visualization, writing – original draft. **Xiran Zhang**: methodology, investigation. **Linwei Li**: methodology. **Xiaomin Yu**: methodology. **Lingbing Zeng**: methodology. **Yanli Cao**: methodology. **Ziwei Tao**: methodology. **Lu Shen**: methodology. **Shuaishuai Zhang**: methodology. **Chuangye Yang**: methodology. **Huizhen Tian**: methodology. **Ying Liang**: methodology. **Hanchen Liao**: methodology. **Xiaotian Huang**: conceptualization, supervision, writing – review and editing. **Qiong Liu**: conceptualization, visualization, supervision, writing – original draft, writing – review and editing.

## Funding

This work was supported by National Natural Science Foundation of China 82203032 and 32260193 (Qiong Liu), Project for high and talent of Science and Technology Innovation in Jiangxi ‘double thousand plan’ jxsq2023301110 (Qiong Liu), Natural Science Foundation of Jiangxi Province 20252BAC250150 (Qiong Liu). The fund from School of Basic Medical Sciences, Nanchang University (Qiong Liu).

## Conflicts of Interest

The authors declare no conflicts of interest.

## Supporting information




**Supporting Information**: jev270274‐sup‐0001‐SuppMat.docx

## Data Availability

The datasets generated during and analysed during the current study are available from the corresponding author on reasonable request.
