# Supplementary material for: An Engineered Nano‐Vesicle Adjuvant Platform (ENAP) for Cytokine Delivery Enables a Novel Antigen‐Coordinated Vaccine Against Helicobacter pylori
Source: J Extracell Vesicles. 2026 Apr 2;15(4):e70274. doi: 10.1002/jev2.70274 (PMC13045921; doi:10.1002/jev2.70274)
Supplement: Supplementary file 1 — Supporting Information: jev270274‐sup‐0001‐SuppMat.docx [file JEV2-15-e70274-s001.docx]

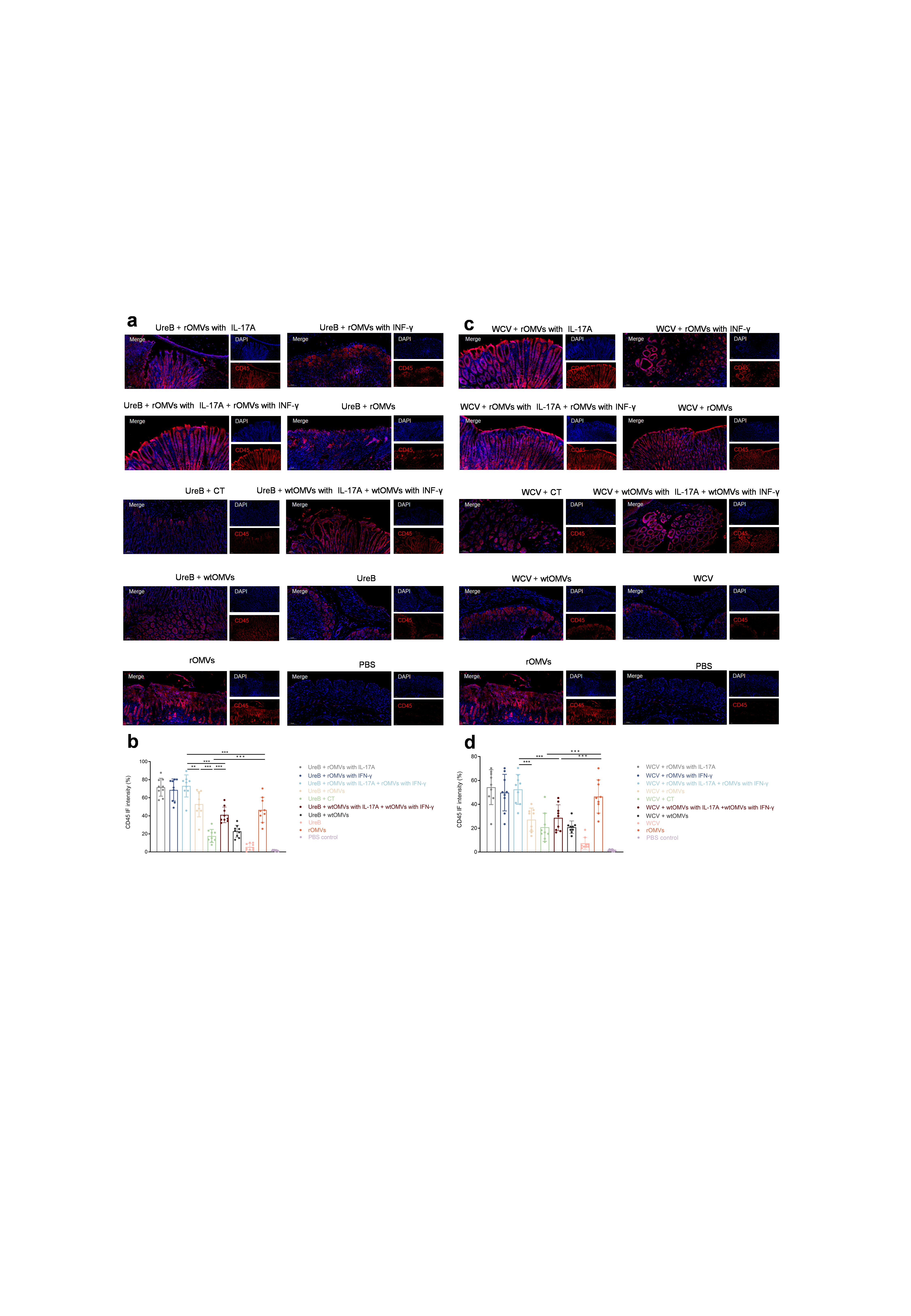


**Figure S1. ENAP promotes the recruitment of CD45⁺ immune cells to the gastric mucosa.** (**a** and **c**) Representative immunofluorescence images of CD45⁺ cells (red) in the gastric mucosa of mice immunized with UreB (a) or WCV (c) antigen combined with the indicated adjuvants. (**b** and **d**) Quantitative analysis of CD45 fluorescence intensity corresponding to panels a and c, respectively. Nuclei were counterstained with DAPI (blue). Data are presented as means ± SD (n = 9 mice per group). Ordinary one-way ANOVA was performed for all comparisons (**P* < 0.05, ***P* < 0.01, ****P* < 0.001).


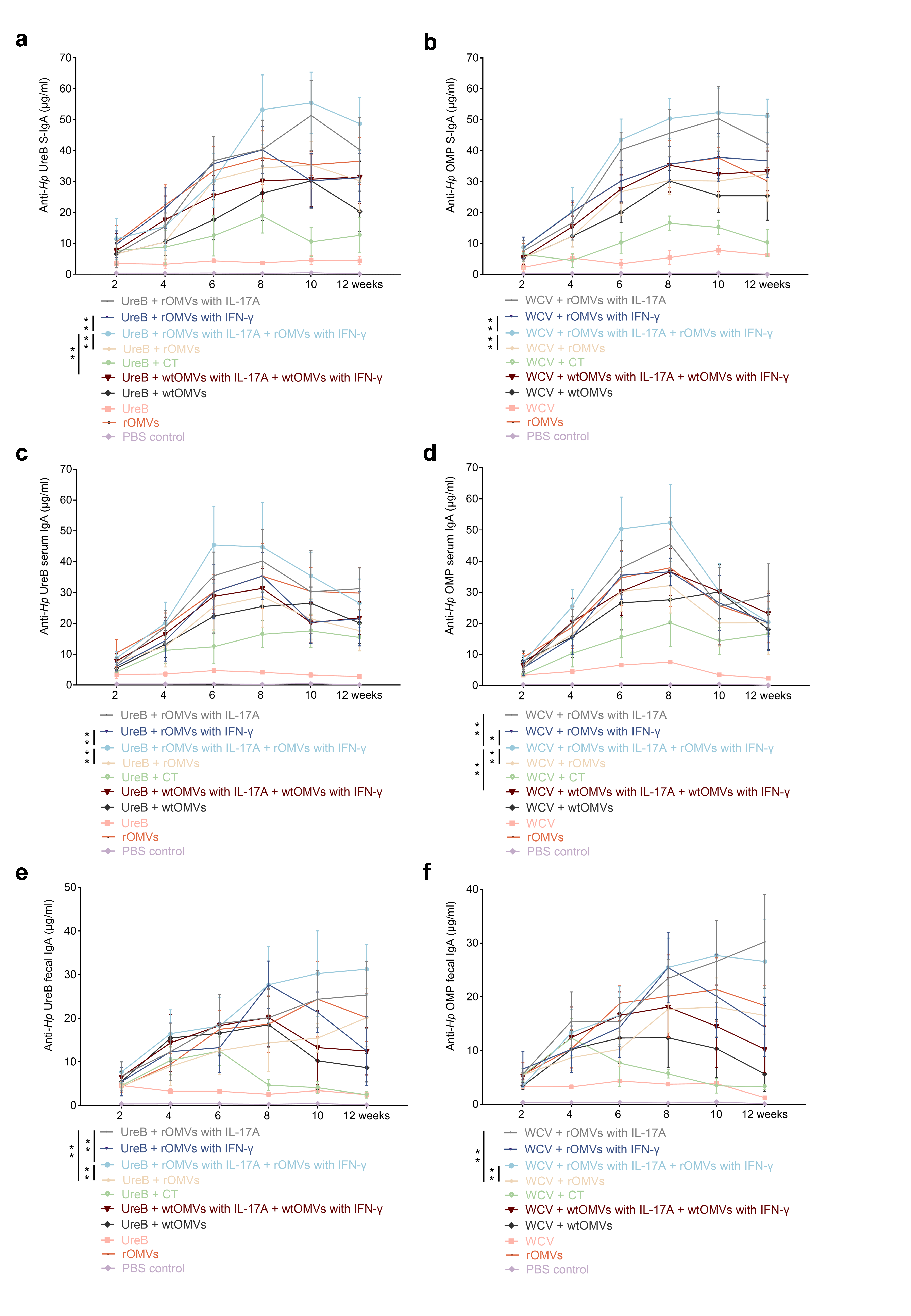


Figure S2. ENAP induces long-lasting mucosal immune responses in mice. (a and b) Longitudinal monitoring of anti-UreB IgA (a) and anti-OMP IgA (b) antibody titers in gastric mucosal extracts over 12 weeks post-immunization, measured by ELISA. (c and d) Longitudinal monitoring of anti-UreB IgA (c) and anti-OMP IgA (d) antibody titers in serum over 12 weeks post-immunization. (e and f) Longitudinal monitoring of anti-UreB IgA (e) and anti-OMP IgA (f) antibody titers in fecal samples over 12 weeks post-immunization. Data are pooled from two independent experiments (n = 9 mice per group) and presented as means ± SD. Ordinary one-way ANOVA was performed for all comparisons (**P* < 0.05, ***P* < 0.01, ****P* < 0.001).


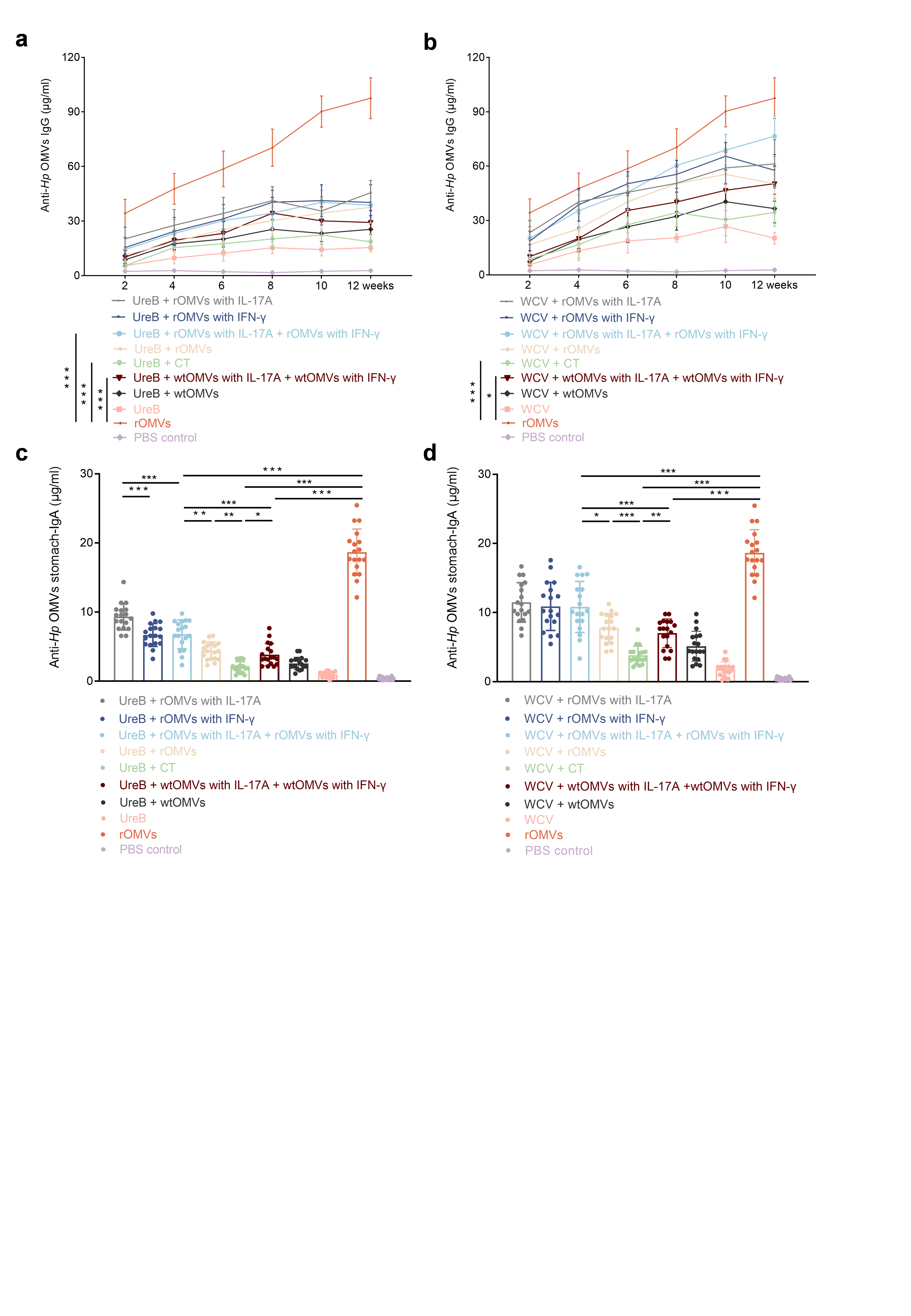


**Figure S3. Cytokine-loaded ENAP elicits antibody responses against the OMV platform.** (**a** and **b**) Longitudinal monitoring of serum anti-OMV IgG titers over 12 weeks after immunization with UreB (a) or WCV (b) as antigens and OMVs as an adjuvant platform, measured by ELISA using purified OMVs as the coating antigen. n = 18 mice (9 per group from two independent experiments); data were pooled for statistical analysis. Statistical comparisons shown are for the endpoint (12-week) titers. (**c** and **d**) Gastric mucosal anti-OMV stomach IgA levels measured by ELISA in stomach homogenates at week 8 post-immunization, using purified OMVs as the coating antigen. Data are pooled from two independent experiments (n = 9 mice per group). Data are presented as means ± SD. Ordinary one-way ANOVA was performed for all comparisons (**P* < 0.05, ***P* < 0.01, ****P* < 0.001).


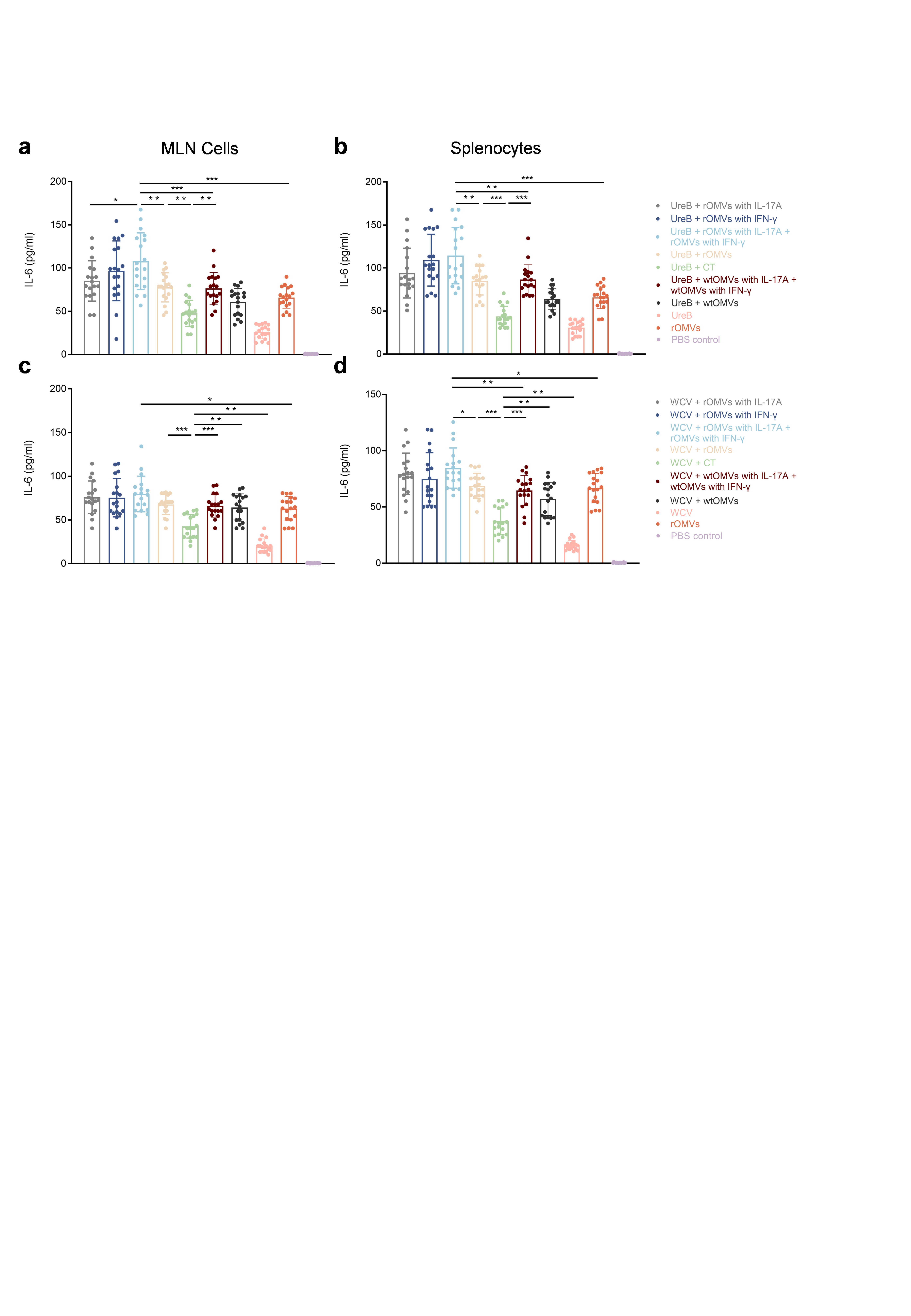


**Figure S4.** **Safety evaluation of the ENAP.** (**a** to **d**) Production of IL-6 measured by ELISA in supernatants from MLN cells (a, c) and splenocytes (b, d) isolated from mice 8 weeks post-immunization with UreB (a, b) or WCV (c, d) antigen combined with ENAP or CT adjuvant, after restimulation with UreB/WCV antigen for 24 h. Data are pooled from two independent experiments (n = 9 mice per group) and presented as means ± SD. Ordinary one-way ANOVA was performed for all comparisons (**P* < 0.05, ***P* < 0.01, ****P* < 0.001).


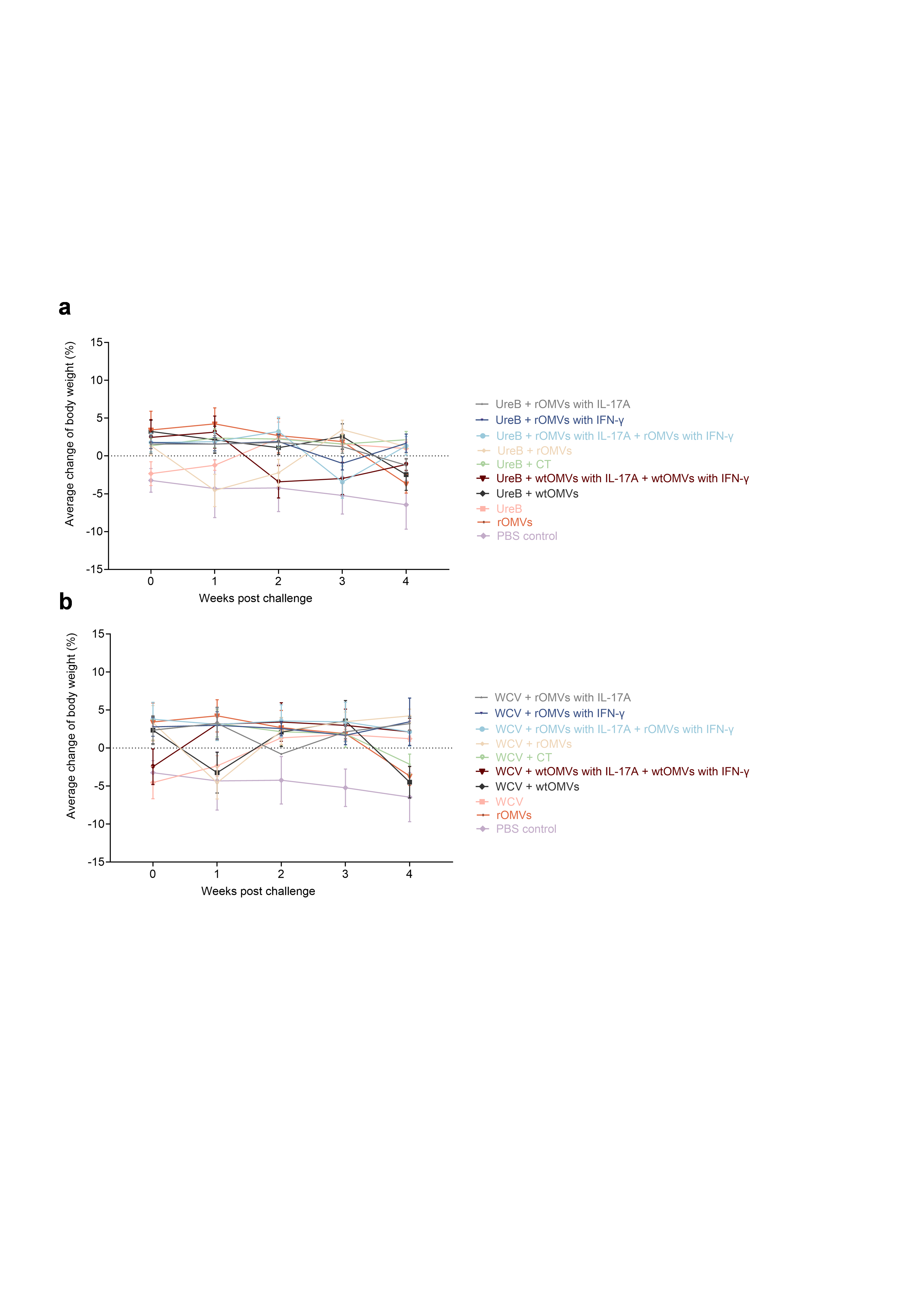


**Figure S5.** **ENAP affects mouse body weight.** (**a** and **b**) Mouse body weights were recorded weekly until 4 weeks after *H. pylori* challenge. Data are from two independent experiments (n = 4 mice per group) and presented as means ± SD.


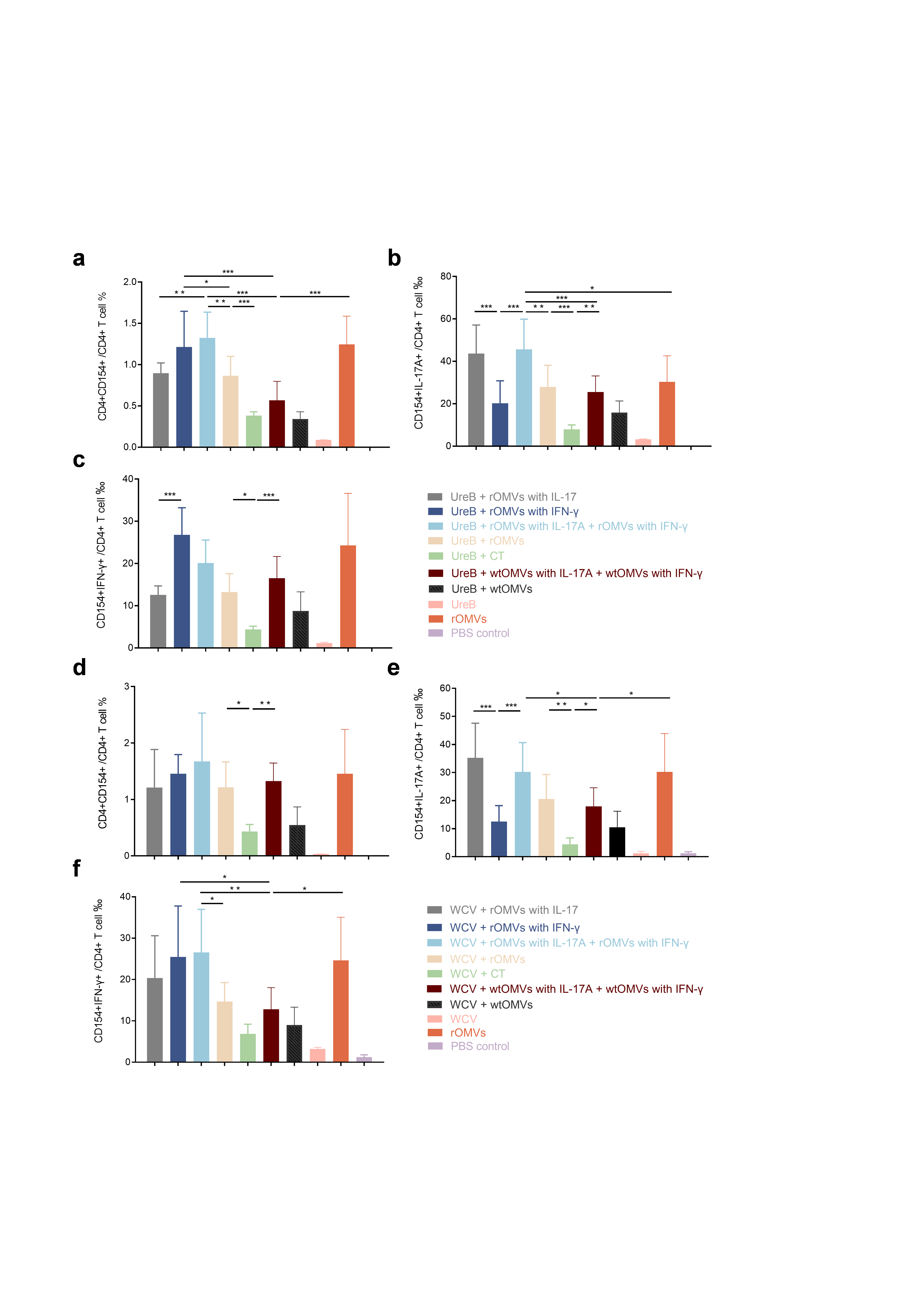


**Figure S6. ENAP elicits Th1 and Th17 cellular immune responses.** (**a** to **f**) Flow cytometric analysis of splenocytes isolated two weeks post-challenge. The percentages of CD4⁺ CD154⁺ T cells (a, d), CD154⁺ IL-17A⁺ T cells (b, e), and CD154⁺ IFN-γ⁺ T cells (c, f) among total CD4⁺ T cells are shown, following intracellular staining for cytokines. Mice were immunized with UreB (a-c) or WCV (d-f) antigen combined with the indicated adjuvants. Data are pooled from two independent experiments (n = 5 mice per group) and presented as means ± SD. Ordinary one-way ANOVA was performed for all comparisons (**P* < 0.05, ***P* < 0.01, ****P* < 0.001).


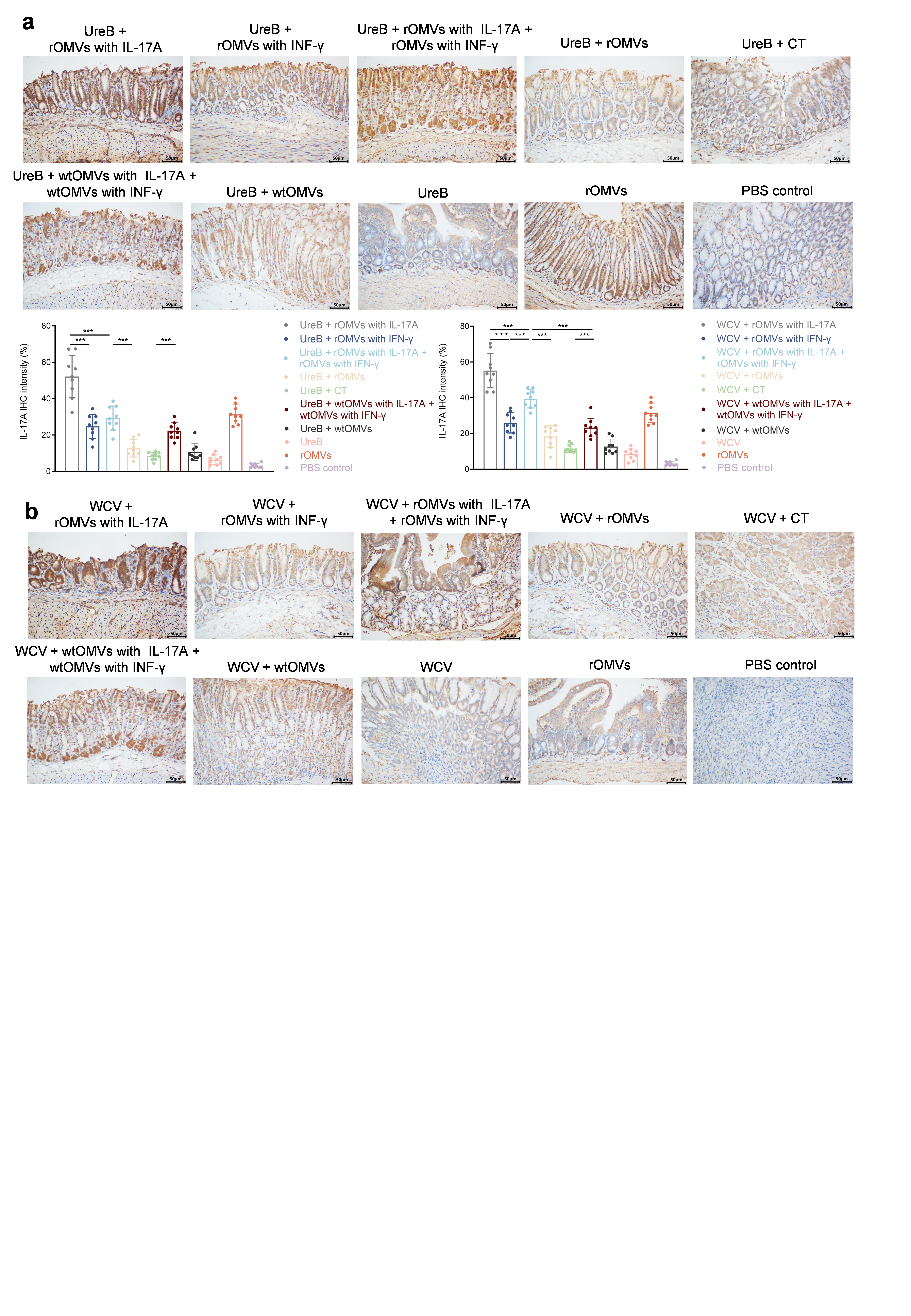


**Figure S7.** **Immunohistochemical analysis of IL-17A expression in gastric mucosa.** (**a** and **b**) Representative immunohistochemical staining images (left) and quantitative analysis (right) of IL-17A expression in gastric mucosal tissues from mice immunized with UreB (a) or WCV (b) antigen combined with the indicated adjuvants. Tissues were collected two weeks post-challenge (day 110). Scale bars, 50 μm. Data are presented as means ± SD (n = 9 mice per group). Ordinary one-way ANOVA was performed for all comparisons (**P* < 0.05, ***P* < 0.01, ****P* < 0.001).


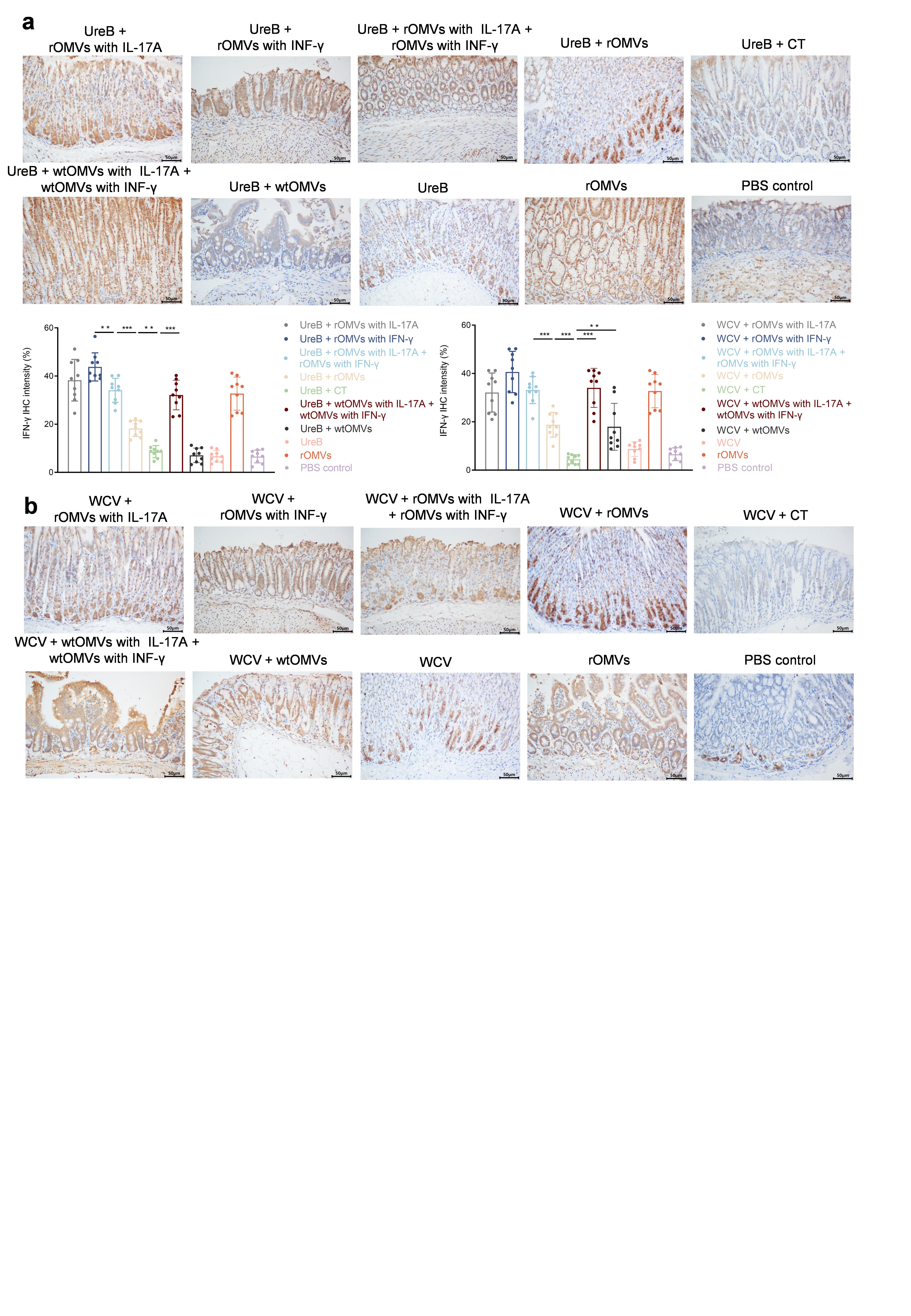


**Figure S8.** **Immunohistochemical analysis of IFN-γ expression in gastric mucosa.** (**a** and **b**) Representative immunohistochemical staining images (left) and quantitative analysis (right) of IFN-γ expression in gastric mucosal tissues from mice immunized with UreB (a) or WCV (b) antigen combined with the indicated adjuvants. Tissues were collected two weeks post-challenge (day 110). Scale bars, 50 μm. Data are presented as means ± SD (n = 9 mice per group). Ordinary one-way ANOVA was performed for all comparisons (**P* < 0.05, ***P* < 0.01, ****P* < 0.001).


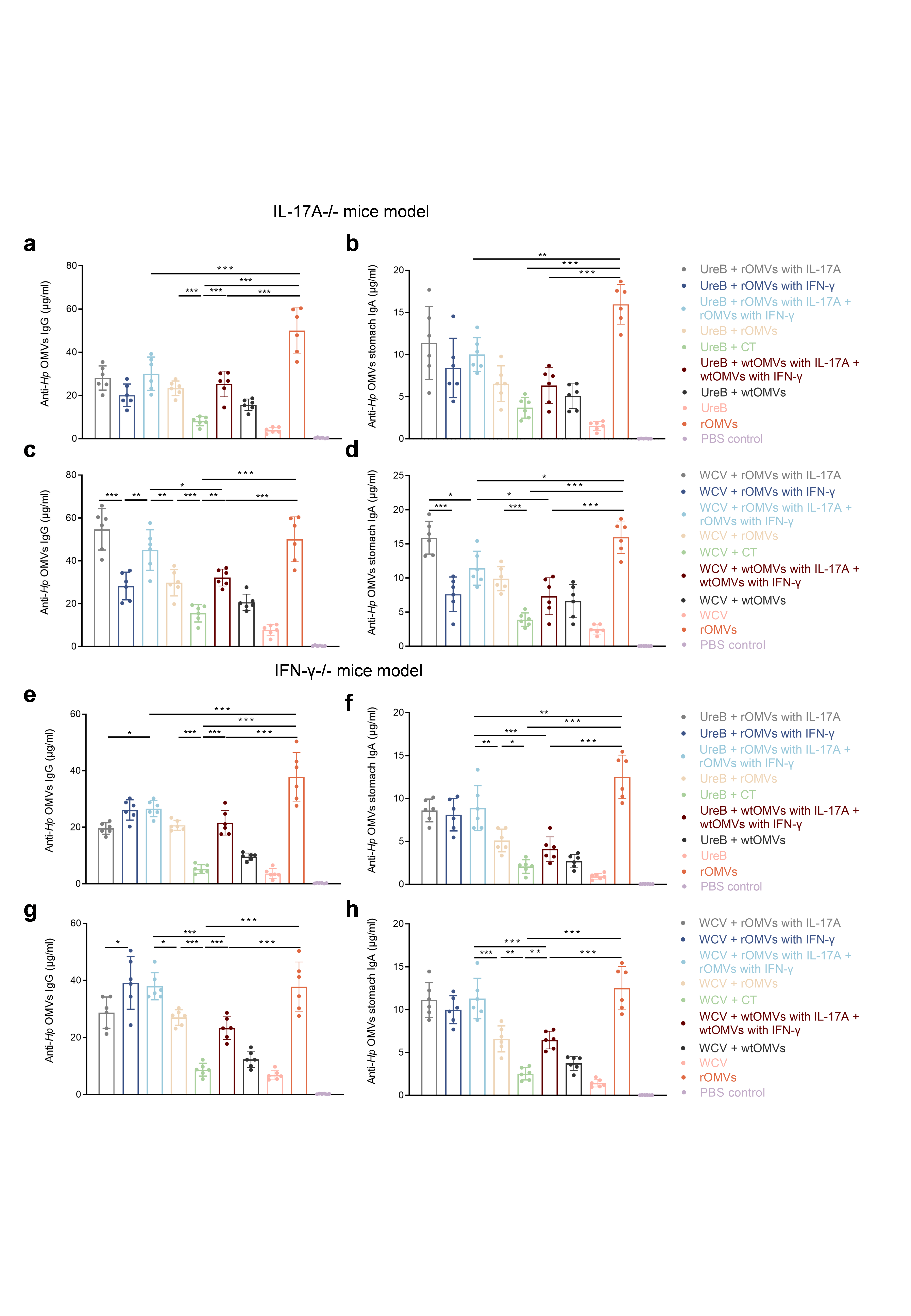


**Figure S9. Anti-OMV antibody responses in IL-17A⁻/⁻ and IFN-γ⁻/⁻ murine models.** (**a**, **c**, **e**, **g**) Serum anti-OMV IgG titers in immunized IL-17A⁻/⁻ (a, c) and IFN-γ⁻/⁻ (e, g) mice, measured by ELISA using purified OMVs as the coating antigen. n = 6 mice per group. (**b**, **d**, **f**, **h**) Gastric mucosal anti-OMV stomach IgA levels in IL-17A⁻/⁻ (b, d) and IFN-γ⁻/⁻ (f, h) mice, measured by ELISA in stomach homogenates at week 8 post-immunization, using purified OMVs as the coating antigen. n = 6 mice per group. Ordinary one-way ANOVA was performed for all comparisons (**P* < 0.05, ***P* < 0.01, ****P* < 0.001).

**Table S1.** Vaccine formulation strategy for immunization using ENAP for cytokine presentation.

| Group (9 mice/each) | Immunogen and Dose (μg/ one mouse) |
| --- | --- |
| 1 | UreB (200) + rOMVs with IL-17A (10) |
| 2 | UreB (200) + rOMVs with INF-γ (10) |
| 3 | UreB (200) + rOMVs with IL-17A (5) + rOMVs with INF-γ (5) |
| 4 | UreB (200) + rOMVs (10) |
| 5 | UreB (200) + wtOMVs with IL-17A (5) + wtOMVs with INF-γ (5) |
| 6 | UreB (200) + wtOMVs (10) |
| 7 | UreB (200) + CT (10) |
| 8 | UreB (200) |
| 9 | WCV + rOMVs with IL-17A (10) |
| 10 | WCV + rOMVs with INF-γ (10) |
| 11 | WCV + rOMVs with IL-17A (5) + rOMVs with INF-γ (5) |
| 12 | WCV + rOMVs (10) |
| 13 | WCV + wtOMVs with IL-17A (5) + wtOMVs with INF-γ (5) |
| 14 | WCV + wtOMVs (10) |
| 15 | WCV + CT (10) |
| 16 | WCV |
| 17 | rOMVs (200) |
| 18 | PBS control |

* All groups used 6-week-old female C57BL/6 mice immunized via the oral route. rOMVs: LPS-modified *H. pylori* OMVs; wtOMVs: wild-type *H. pylori* OMVs.
